# Electrochemical Nitrogen Fixation Using CeFeO_3_ and CeO_2_ for Ammonia Synthesis and Nitrate Remediation

**DOI:** 10.1021/acsami.5c07123

**Published:** 2025-06-12

**Authors:** James Ebenezer, Parthiban Velayudham, Alex Schechter

**Affiliations:** † Department of Chemical Sciences, 42732 Ariel University, Ariel 40 700, Israel; ‡ Research and Development Centre for Renewable Energy, New Technology Centre, University of West Bohemia, 301 00 Pilsen, Czech Republic

**Keywords:** orthoferrite, nitrate remediation, green ammonia, H_2_−NO_3_^−^ fuel cell

## Abstract

In the pursuit of sustainable ammonia synthesis and nitrate remediation, electrochemical nitrate reduction to ammonia (eNO_3_RR) emerges as a promising alternative to the carbon-intensive Haber–Bosch process, which emits 1.6–2.0 tons of CO_2_ per ton of ammonia. Powered by renewable energy, the eNO_3_RR offers reduced emissions and energy consumption but faces challenges in catalytic activity and product selectivity due to its complex mechanism. To address these issues, CeFeO_3_ supported CeO_2_ composites were synthesized via a microwave polyol method with varying Ce:Fe atomic ratios and comprehensively characterized. Electrochemical analysis revealed that pure CeO_2_ achieved a high ammonia yield rate of 4040.5 ± 262.5 μg h^–1^ cm^–2^ but with a lower Faradaic efficiency (FE) of 52.8 ± 2.8% at −0.45 V_RHE_ in 0.1 M KOH with 0.1 M NO_3_^–^. Introducing CeFeO_3_ into CeO_2_ enhanced FE significantly, reaching a maximum of 80.1 ± 3.3% with an ammonia yield rate of 3223.9 ± 168.3 μg h^–1^ cm^–2^. Parasitic hydrogen evolution accounted for only 4.9 ± 0.9% FE, while hydroxylamine and nitrite, key intermediates, contributed 8.3 ± 1.2% and 6.7 ± 0.9%, respectively. Stability was demonstrated over 25 one hour cycles (25 h total) at −0.45 V_RHE_ with electrolyte replacement. The intrinsic perovskite structure of CeFeO_3_, facilitating electron exchange via oxygen vacancies, underpinned the improved performance. H_2_–NO_3_^–^ fuel cell studies showed 74.6% thermodynamic efficiency at a current density of 29.7 mA cm^–2^ at 0.46 V. This study underscores CeFeO_3_/CeO_2_ composites’ potential for sustainable ammonia production and environmental remediation.

## Graphical abstract

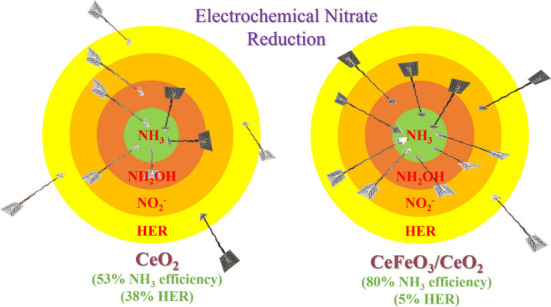

## 1 Introduction

Electrochemical ammonia synthesis from nitrogen (eNRR) is an innovative approach aimed at producing ammonia gas as an alternative CO_2_ free hydrogen carrier.^1^ Unlike the traditional Haber–Bosch process, which emits about 1.6 to 2.0 tons of CO_2_ per ton of ammonia, the eNRR process utilizes water and renewable resources to chemically store energy.^2^ Nevertheless, the very high activation energy barrier of N_2_ required to cleave N≡N bond (940 kJ mol^–1^), very low aqueous solubility of N_2_, and poor selectivity due to undesired dominating hydrogen evolution reaction (HER) makes the eNRR process impractical for large scale energy storage. These inefficiencies hinder the commercialization despite more than a decade of research and development efforts.^3^ On the other hand, nitrate, a known contaminant in many water bodies, resulting from agricultural runoff and industrial wastes, is considered as an alternative nitrogen source for N_2_ in electrochemical ammonia production.^4^ The eight-electron reduction of NO_3_^–^ to NH_3_ is known as the electrochemical nitrate reduction reaction (eNO_3_RR).^5,6^

1 $$\begin{array}{c} {{\mathrm{NO}}_{3}}^{-}+6H_{2}O+8e^{-}\to {\mathrm{NH}}_{3}+9{\mathrm{OH}}^{-}\\ E^{0}=-0.12\;\mathrm{V vs}.\mathrm{SHE} \end{array}$$

The high solubility of nitrate ions in water, 880 g L^–1^ at 20 °C compare to 20 mg L^–1^ of N_2_, makes them more reactive and accessible than N_2_ gas. Additionally, the low dissociation energy of 240 kJ mol^–1^ of the N=O bond of nitrates is also an advantage over that of N_2_ in view of the activation barrier. This results in faster kinetics and greater selectivity toward ammonia rather than hydrogen. Despite these advantages, the eNO_3_RR involves a complex eight-electron transfer process depending on the electrolyte pH, as illustrated in the Frost–Ebsworth diagram in Figure 1. In alkaline conditions, several possible key stable intermediates can be identified, which include nitrite (via a 2e^–^ reduction product) and hydroxylamine (a 4e^–^ reduction product of NO_2_^–^). Hydroxylamine (NH_2_OH) can then be further reduced by 2e^–^ transfer to form ammonia. This complexity underscores the necessity for specialized catalysts that are highly selective toward ammonia production.

**Figure 1:**
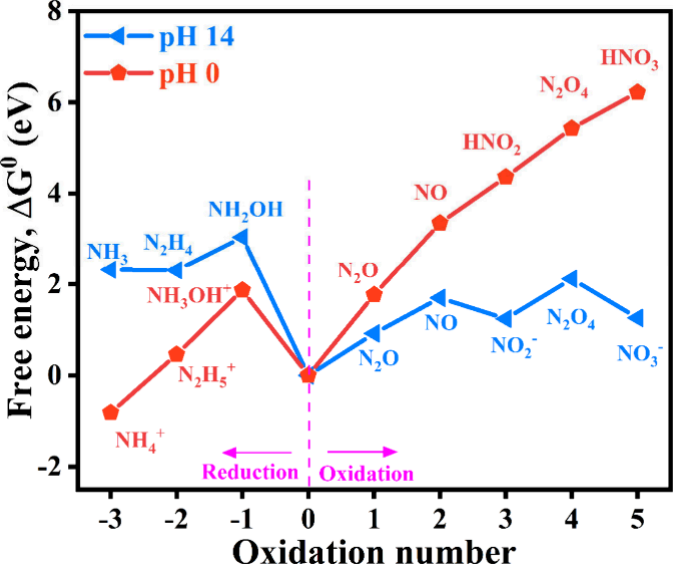
Frost–Ebsworth diagram of nitrogen species at pH 0.0 (red) and pH 14.0 (blue).^7^

Rare earth orthoferrites, characterized by their distinctive perovskite structure (RFeO_3_, where R represents a rare earth element), present several benefits. These include effective photocatalysis under visible light, robust chemical stability, and multifunctionality due to their multiferroic properties.^8,9^ The multiferroicity enhances the magnetic recovery of the photocatalyst and the ferroelectric characteristics contribute to elevated photocatalytic and electrocatalytic performance in catalysis applications.^10^ Their chemical and thermal stabilities ensure long-term durability under various electrochemical conditions. Adjusting rare earth elements optimizes the electronic conductivity and band gap. Oxygen vacancies from surface defects improve performance in oxygen evolution and reduction reactions.^11^ This adaptability is suitable for energy conversion and environmental remediation applications of rare earth elements.

Among the rare earth oxides, cerium oxide (CeO_2_) stands out for its superior reducibility, excellent electronic/ionic conductivity, and varied morphology, showing significant advantages in electrocatalysis, especially in reduction reactions.^12–16^ To enhance the eNO_3_RR performance, cerium has been doped or composited with other metals or metal oxides. For instance, Luo et al. reported that Ce-doped MoS_2–*x*_ nanoflower arrays achieved 96.6% NH_3_ Faradaic efficiency (FE) and 7.3 mg h^–1^ cm^–2^ yield rate at −0.7 V vs RHE.^17^ The authors emphasize that doping with cerium (Ce) leads to the formation of sulfur vacancies (V_s_). These vacancies, along with better NO_3_^–^ activation, effectively reduce the thermodynamic energy barrier of the *NO → *NOH step, which is a crucial rate-determining step in the reaction process. Furthermore, Yang et al. explored the impact of Ce content in Ce-doped Cu electrodes for eNO_3_RR.^18^ In 1 M KOH electrolyte with 1400 ppm nitrate-N, the Cu_10_Ce_10_ electrode demonstrated an ammonia yield rate of 1.0 mmol h^–1^ cm^–2^ and 98.4% FE at −1.34 V vs RHE. This high efficiency was attributed to the formation of Ce^3+^ which leads to increased Cu^2+^ content via Ce doping, leading to the formation of an active Cu/Cu_2_O interfacial structure. In addition, CeO_*x*_ also serves as effective support for anchoring electrocatalysts via shared oxygen surface atoms.^19^ Lv et al. reported that CeO_2_ triggered the band alignment for charge transfer in nitrogen reduction with Bi_4_V_2_O_11_/CeO_2_ with 23.2 μg h^–1^ mg_cat_^–1^ ammonia yield rate and 10% FE at −0.2 V vs RHE.^20^ The CeO_2_ support 5 times enhanced the ammonia rate compared to Bi_4_V_2_O_11_ alone. Likewise, Au nanoparticles were also supported on CeO_*x*_-RGO bisubstrate for nitrogen reduction. Li et al. also confirmed the effective charge transfer in the presence of CeO_*x*_.^21^ Regarding orthoferrites, bismuth ferrite (BiFeO_3_) was specifically studied for nitrate reduction by Wang et al.^22^ Authors reported 90.4 mg h^–1^ mg_cat_^–1^ ammonia yield rate with 96.8% efficiency at −0.6 V vs RHE in 0.1 M KOH containing 0.1 M KNO_3_ solution. The long-term stability of the catalyst was attributed to the transformation of its distorted-perovskite-type structure into an amorphous phase during nitrate reduction.

In this context, we designed a rare-earth orthoferrite catalyst (CeFeO_3_), supported on its own oxide phase (CeO_2_), to enhance the electrochemical nitrate reduction reaction under alkaline conditions. This self-supported hybrid structure exploits the cooperative interaction between CeFeO_3_ and CeO_2_, modulating the electronic environment, increasing the exposure of active sites, and effectively suppressing the competing HER, a key limitation in many conventional transition-metal-based or single-phase catalyst oxide materials. As a result, the CeFeO_3_/CeO_2_ composite exhibited a remarkable ammonia yield rate of 3223.9 ± 168.3 μg h^–1^ cm^–2^ and a FE of 80.1 ± 3.3% at −0.45 V vs RHE. In contrast, the CeO_2_ support alone achieved a slightly higher yield rate of 4040.5 ± 262.5 μg h^–1^ cm^–2^ but with a significantly lower FE of 52.8 ± 2.8%, highlighting the critical role of CeFeO_3_ in enhancing selectivity toward ammonia formation. The incorporation of CeFeO_3_ nearly doubled the FE by effectively mitigating the HER. Moreover, the optimized CeFeO_3_/CeO_2_ catalyst demonstrated excellent durability, maintaining consistent electrochemical performance over 25 consecutive eNO_3_RR cycles at the same potential. This combination of high activity, selectivity, and stability underscores the potential of rare-earth orthoferrite-based hybrid systems as robust and sustainable electrocatalysts for ammonia production.

## 2 Experimental Section

### 2.1 Materials

Cerium­(III) nitrate hexahydrate (99.9+% Ce) and iron­(III) nitrate nonahydrate (98+%) were acquired from Strem Chemical Inc. Poly­(vinyl alcohol) (PVA) along with potassium hydroxide, sodium citrate dihydrate (over 99 wt %), sodium nitroprusside dihydrate, ammonium chloride, and salicylic acid were sourced from Sigma-Aldrich. Sulfuric acid (95–98 wt %), isopropyl alcohol, and sodium hypochlorite solution (containing 11–15 wt % available chlorine) were procured from Honeywell, Bio-Lab, Ltd. (Jerusalem) and Thermo Scientific, respectively. Vulcan XC-72 carbon was obtained from Cabot Corporation. Both the Nafion 115,117 membrane and Nafion ionomer (a 5 wt % solution in a mix of lower aliphatic alcohols and water) were purchased from the Fuel Cell Store. Ultra-high-purity water with resistivity of 18 MΩ cm was used for all experiments. All chemicals were used in their received state without any further processing.

### 2.2 Synthesis of CeFeO_3_/CeO_2_ Composite Catalyst

The synthesis of CeFeO_3_/CeO_2_ composites was carried out using a polyol-assisted microwave method (shown in Scheme 1), chosen for its inherent polarity that facilitates rapid and localized heating at the molecular level.^23^ The poly­(vinyl alcohol) (PVA) was used as a capping agent to polydisperse Ce and Fe uniformly throughput the composite mixture.^24^ An aqueous solution of PVA (MW = 9000–10,000) was prepared by dissolving 4 g of PVA in 96 mL of deionized water at 80 °C. Different molar ratios of iron nitrate and cerium nitrate were added to a specific volume of water, mixed well, and added to the polymer solution, followed by continuous stirring for 2 h with a magnetic stirrer. This mixture was microwave treated at 1000 W power for 30 min until complete evaporation of water. The resultant dried solid was washed with water until no more nitrate content was detected in the UV–visible analysis. Resultant powder was calcined under Ar atmosphere at 400 °C for 3 h and thus resulted a desired composite CeFeO_3_/CeO_2_ catalysts. Pure cerium oxide and iron oxide catalysts were synthesized using the same methodology without the addition of iron nitrate or cerium nitrate salt.

**Scheme 1:**
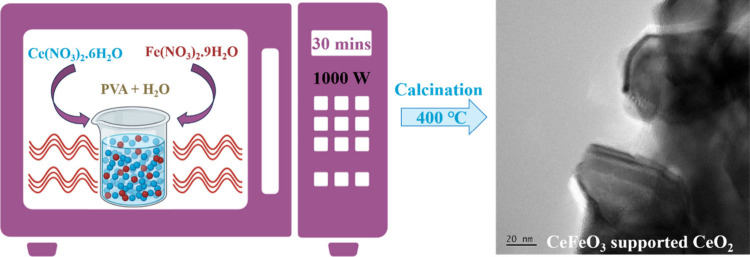
Schematic Representation of CeFeO_3_ Supported CeO_2_ Synthesis

### 2.3 Material Characterizations

Powder X-ray diffraction (XRD) analysis of the samples was performed by using a Rigaku Smart Lab SE diffractometer. The Raman spectroscopic analysis was conducted using a Kratos AXIS-HS spectrometer with a 532 nm laser and a monochromatized Al Kα source as well as the XploRA ONE micro-Raman system from Horiba Scientific, Japan. To determine the iron content in the electrolyte, inductively coupled plasma–optical emission spectroscopy (ICP-OES) was carried out using a Spectro Arcos optical emission spectrometer. For X-ray photoelectron spectroscopy (XPS) analysis, which provides insight into the electronic/chemical states and elemental composition of the catalyst, a Nexsa spectrometer equipped with a monochromatic Al Kα X-ray source was used. The MAIA3 TESCAN model scanning electron microscope (SEM) was utilized for the morphological analysis of the materials. High-resolution transmission electron microscopy (HR-TEM) and selected area electron diffraction (SAED) analyses were carried out using a JEOL JEM 2200FS instrument. The samples were prepared by depositing them onto perforated carbon-coated copper grids followed by drying prior to measurement. The N_2_ temperature-programmed desorption (TPD) and H_2_ temperature-programmed reduction (TPR) analyses were done using a Micrometrics AutoChem II chemisorption analyzer coupled with Hiden analytical HPR-20 QIC benchtop gas analysis system. Finally, 150 mg of synthesized composite were stored in the Micromeritics Vacprep 061 sample degassing equipment overnight and the Micromeritics TriStar TM PLUS was used to quantify the BET surface area and pores.

### 2.4 Electrochemical Measurements

All of the electrochemical measurements were conducted in a double chamber H-type cell using a standard three-electrode configuration. A mercury/mercury oxide (Hg/HgO) electrode served as the reference, while a nickel strip (with 99.9% purity) was employed as the counter electrode. To prepare the working electrode, 80 wt % of the CeFeO_3_/CeO_2_ composite catalyst was mixed with 20 wt % Vulcan carbon XC-72. This admixture was then added to 2 mL of a 1:1 isopropanol (IPA):water solution and 20 wt % Nafion ionomer. The resulting mixture was subjected to sonication for 30 min to ensure homogeneity. The homogenized catalyst dispersion was then drop-cast onto Teflonized Toray carbon paper with an active area of 1 × 1 cm^2^. After drying, this paper was used as the working electrode, maintaining a total catalyst (catalyst + Vulcan carbon) loading of 2 mg cm^–2^ for all experiments. The electrochemical tests were carried out using a BioLogic potentiostat/galvanostat workstation (VSP/VMP 3B-20). The electrochemical nitrate reduction reaction activity of the catalyst was assessed in an argon-saturated 0.1 M KOH electrolyte containing 0.1 M KNO_3_ (35.0 mL). The 0.1 M KOH solution was used as an anolyte. Pretreated Nafion115 membrane was used as a separator. Argon gas was purged for 30 min prior to each experiment. To collect ammonia, a 1 mM H_2_SO_4_ acid trap (10 mL) was connected to an outlet of the electrochemical cell. All potentials reported were referenced against the reversible hydrogen electrode (RHE), with pH adjustments made using the Nernst equation, as described in eq 2.

2 $$E_{\mathrm{RHE}}=E_{\mathrm{measured}}+E_{\mathrm{Hg}/\mathrm{HgO}}+\left(0.059\times \mathrm{pH}\right)$$

### 2.5 Nitrate/Nitrite Quantification

The nitrate/nitrite concentration in the electrolyte was determined by using a UV–visible spectrophotometer (Thermo Scientific Pro). The absorbance at 300 and 350 nm was measured for nitrate (NO_3_^–^) and nitrite (NO_2_^–^), respectively.^6^ A calibration curve, based on known nitrate/nitrite concentrations in 0.1 M KOH solution, is presented in Figures S1 and S2. The rate of nitrite formation and its Faradaic efficiency were calculated by using eqs 3 and 4.

$${{\mathrm{NO}}_{2}}^{-}\;\mathrm{formation rate}=\frac{C\times V}{t\times A}$$ 3

$$\mathrm{FE}\%=\frac{n\times F\times C\times V}{Q\times \mathrm{MW}}$$ 4

where *C* is the concentration of NO_2_^–^ in μM; *V* is sample volume in mL; *t* is electrolysis time in hours; *A* is the electrode active area in cm^2^; *Q* is applied charge in coulombs; *n* is number of electron transfer (2 e^–^); MW is the molecular weight of NO_2_^–^ (46.005 g mol^–1^); and *F* is the Faraday constant of 96,485 C mol^–1^.

### 2.6 Ammonia Quantification

Ammonia concentration in the samples was determined using a UV–visible spectrophotometer, employing the indophenol blue method.^21^ Initially, approximately 200 μL of each sample was diluted to a final volume of 2.0 mL. This dilution was performed using a 0.1 M potassium hydroxide (KOH) solution, which contained 5 wt % salicylic acid and 5 wt % sodium citrate dissolved in a 1 M sodium hydroxide (NaOH) solution. In the subsequent step, 1 mL of 0.05 M sodium hypochlorite (NaOCl) followed by 0.2 mL of 1 wt % sodium nitroprusside (Na_2_[Fe­(CN)_5_NO]) were added to the mixture. The reaction mixture was then stored in a dark place for 1 h to ensure the completion of the reaction before measuring the absorbance at 650 nm. For calibration purposes, a series of ammonia standards with known concentrations were prepared in a 0.1 M KOH solution. These standards were subjected to the same procedure to construct a calibration curve, which is presented in Figure S3. The ammonia ion selective electrode (Thermo Scientific, model no. 9512HPBNWP) was utilized to validate the indophenol quantification method. Initially, the electrode was calibrated using ammonia standards of 0.1, 1.0, and 10.0 ppm (Figure S4c), followed by the analysis of electrolyte samples. The ammonia formation rate and Faradaic efficiency were calculated using eqs 3 and 4, respectively. These calculations incorporated the molecular weight of ammonia, which is 17.031 g mol^–1^, and the number of electron transfer is 8.

### 2.7 Hydroxylamine Quantification

The concentration of hydroxylamine (NH_2_OH) was determined using a modified colorimetric method adapted from an established protocol reported elsewhere.^26^ Standard calibration curves was developed from known selected standard solutions of NH_2_OH in 0.1 M KOH and is depicted in Figure S4. The yield rate and efficiency were calculated using eqs 3 and 4 incorporating the molecular weight of NH_2_OH (33.034 g mol^−1^) and the number of electrons as 6.

## 3 Results and Discussion

### 3.1 Physico-Chemical Characterization of CeFeO_3_/CeO_2_ Composite Catalysts

The structural characterizations of the synthesized CeFeO_3_/CeO_2_ composite catalyst were analyzed by XRD and Raman spectroscopy as illustrated in Figure 2. The cerium oxide support (Figure 2a) shows characteristic diffraction peaks at 2θ values of 28.5°, 33.1°, 47.4°, 56.3°, 59.1°, 69.4°, 76.7°, 79.1°, and 88.4°, corresponding to the (111), (200), (311), (222), (400), (331), (420), and (422) planes of a cubic structure (JCPDS 00-034-0394),^27^ respectively. The successful support of CeFeO_3_ on CeO_2_ confirmed by the orthorhombic phase (JCPDS 00-022-0166)^27,28^ at 2θ values of 22.7°, 32.3°, 39.8°, 46.3°, 57.8°, and 67.6°, which align with the (110), (112), (202), (004), (024), and (224) planes in Figure 2b. Also, the enhancement of CeFeO_3_ phase reflections were observed on increasing the Fe atomic ratio in CeO_2_ shown in Figure S5a. The partially enlarged spectrum in Figure S5b exhibits a shift in the (111) plane of CeO_2_ to larger angles at high Fe contents. This shift likely indicates the formation of A-site deficiencies in the crystal structure, causing the lattice to contract as the Fe concentration rises.^29^ To further understand the structural distortion, the average micro strain (ε) indication of lattice distortion due to imperfections or defects was calculated using the following eq 5.^30^

5 $$\mathrm{micro strain}\;\left(\varepsilon \right)=\frac{\beta \cos\theta }{4}$$

where β is the fwhm and θ is the diffraction angle.

**Figure 2:**
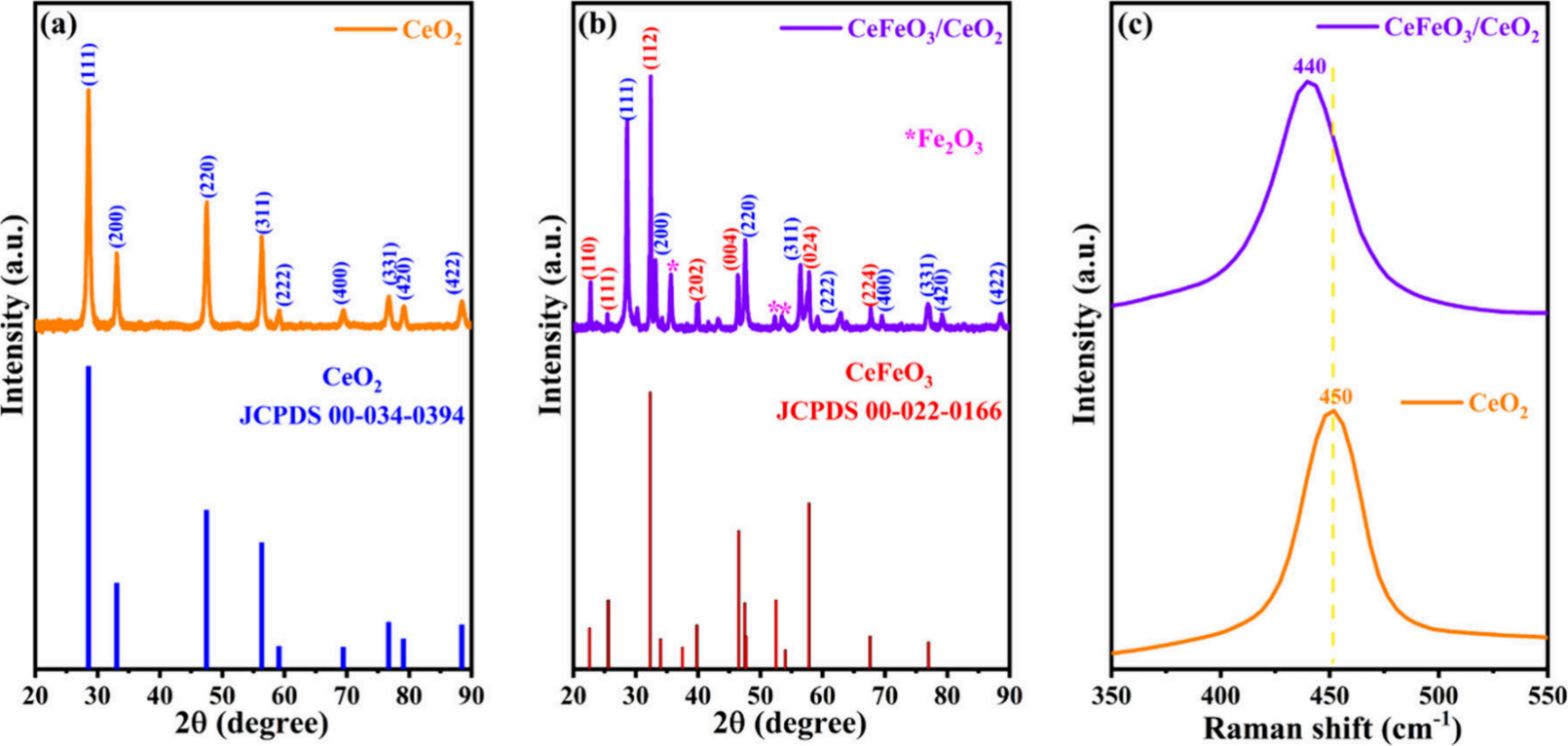
X-ray diffraction patterns of (a) CeO_2_ and (b) CeFeO_3_ supported on CeO_2_ catalysts; (c) Raman spectra of CeO_2_ and CeFeO_3_ supported on CeO_2_ catalysts.

For pure CeO_2_, the calculated micro strain was 11.6 × 10^3^. Upon the addition of 5% Fe, the strain decreased significantly to 3.0 × 10^3^, indicating a relaxation of the lattice due to low level CeFeO_3_ formation (Table S1). However, with further Fe addition (25% and 50%), the micro strain increased to 7.3 × 10^3^ and 8.5 × 10^3^, respectively. This increase is likely due to excessive lattice distortion and mismatch arising from higher CeFeO_3_ incorporation, which disrupts the crystal symmetry of the CeO_2_ support and introduces localized strain fields. The crystallite size of CeO_2_ was also calculated using the Scherrer equation, yielding an average of 20.4 nm for the (111) plane. Upon CeFeO_3_ loading, the crystallite size increased progressively with Fe content as 30.0 nm (5% Fe), 65.7 nm (25% Fe), and 78.3 nm (50% Fe). For the CeFeO_3_ phase (evaluated using the (112) plane), the corresponding crystallite sizes were 124.8, 196.4, and 216.8 nm for 5%, 25%, and 50% Fe compositions, respectively, reflecting significant grain growth at higher Fe loadings.

The Raman spectroscopy study, illustrated in Figure 2c, investigated the impact of CeFeO_3_ formation on the CeO_2_ support. For CeO_2_, a Raman shift was observed at 450 cm^–1^, originating from the T_2g_ symmetric vibration of the Ce–O–Ce bond.^31^ The formation of the CeFeO_3_ phase shifts the Raman band negatively (Figure S5c), indicating local lattice imperfections caused by the synergistic effect of CeFeO_3_ within the CeO_2_ support.^32^ The scanning electron microscopy (SEM) micrographs of the as-synthesized catalysts, displayed in Figure S6, reveal a sponge-like morphology across all composite catalysts. The inclusion of PVA in synthesis as a capping agent ensures uniform particle dispersion and stabilization, contributing to the composite’s unique morphology. The homogeneous distribution of metal oxides within the composite is confirmed by energy-dispersive X-ray spectroscopy (EDS) mapping, as shown in Figure S7. EDS elemental analysis of CeO_2_ shows 84.5% Ce and 15.5% O, closely matching the theoretical composition of 81.4% Ce and 18.6% O. Additionally, the introduction of CeFeO_3_ results in compositions of 56.9% Ce, 32.1% Fe, and 20.1% O. The BET surface area measurements of the catalysts were also investigated (Figure S8). CeO_2_ exhibited a specific surface area of 11.75 m^2^ g^–1^ with an average pore size of 17.20 nm. However, supporting CeFeO_3_ on CeO_2_ resulted in a significantly lower specific surface area of 1.86 m^2^ g^–1^, despite a reduction in average pore size to 10.27 nm. This decrease in the surface area and pore size can be attributed to the filling of pores and partial coverage of the CeO_2_ surface by the CeFeO_3_ phase, which reduces the available surface for physical adsorption and alters the porosity. The morphology of the CeFeO_3_ supported CeO_2_ catalyst was further examined using high-resolution transmission electron microscopy (HR-TEM), as depicted in Figures 3b, 3d, and 3f. The HR-TEM images confirm the material’s high crystallinity, showing two distinct lattice fringes with *d*-spacing values of 0.216 and 0.142 nm. These values correspond to the *d*-spacing calculated from XRD: 0.210 nm for the (111) plane of CeO_2_ and 0.138 nm for the (112) plane of CeFeO_3_. EDS analysis of the specific region further corroborates the presence of CeO_2_ and CeFeO_3_ phases (Table S2). The lattice fringe of the (202) plane (0.238 nm) of CeFeO_3_ was also confirmed in HR-TEM analysis (Figure 3f). These results confirm the formation of a CeFeO_3_ phase supported on a CeO_2_ matrix. Additionally, the selected area electron diffraction (SAED) pattern, shown in Figures 3c and 3e, was recorded to investigate the crystal structure of the catalyst. The SAED pattern reveals several Debye–Scherrer diffraction rings, which are indexed to the crystal planes of CeFeO_3_ (Figure 3e) and CeO_2_ (Figure 3c), further verifying the crystalline nature of the material.

**Figure 3:**
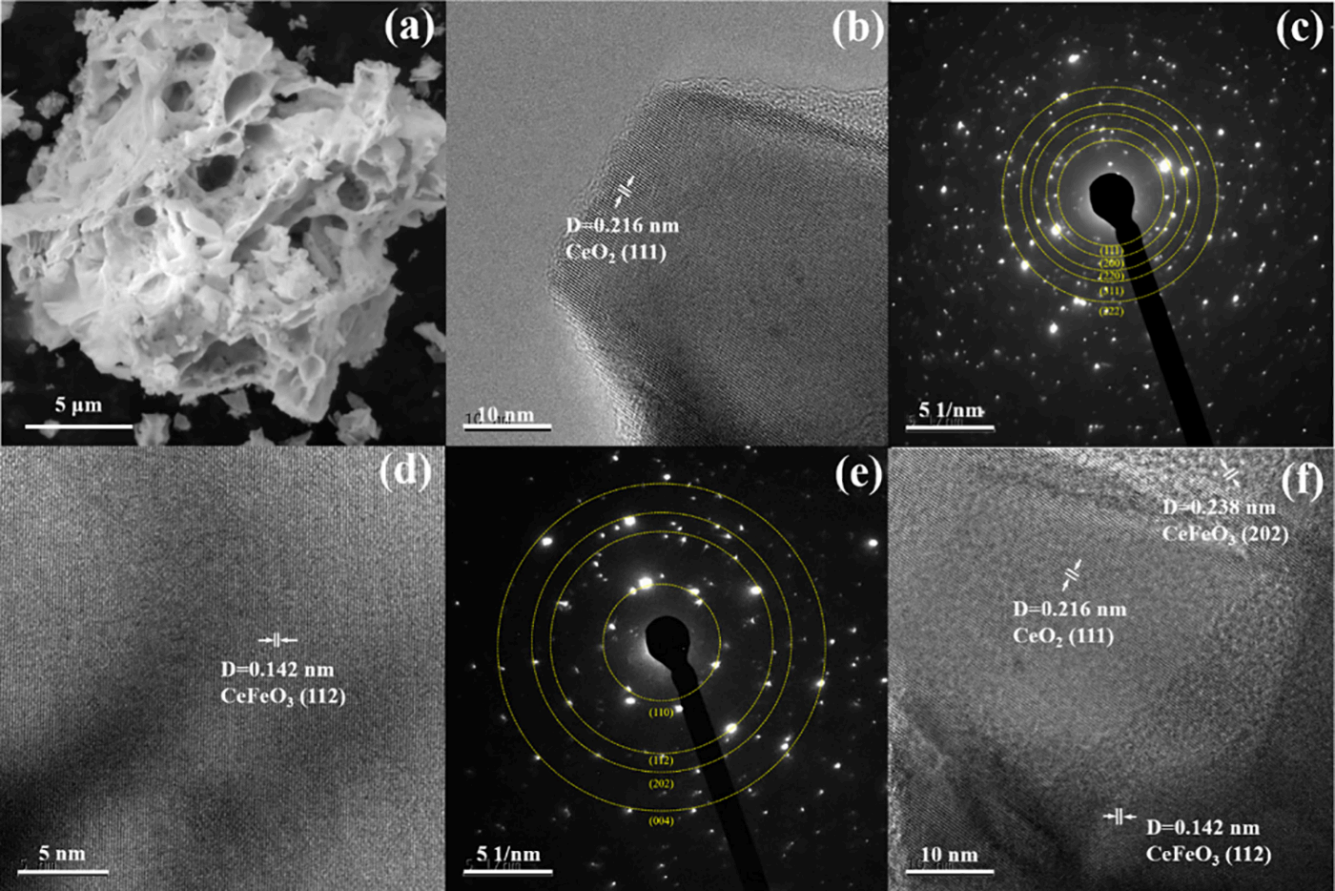
(a) Scanning electron microscopy image; (b) high resolution transmission electron microscopic images of CeO_2_; (c) corresponding selected area diffraction pattern; (d and e) CeFeO_3_ supported CeO_2_ at different spots; and (f) selected area diffraction pattern of CeFeO_3_.

The elemental composition and chemical state of the sample were elucidated through X-ray photoelectron spectroscopy (XPS) analysis. The survey spectrum of CeFeO_3_/CeO_2_, shown in Figure 4a, highlights the presence of cerium (Ce), iron (Fe), and oxygen (O). Additionally, the spectrum includes specific peaks, which are further deconvoluted and detailed in Figures 4b–4d. Notably, peaks at 284.80 and 407.14 eV are attributed to carbon (C 1s)^6,33^ and nitrogen (N 1s),^34,35^ respectively. The carbon peak is associated with poly­(vinyl alcohol) (PVA) remains, while the nitrogen peak results from the use of nitrate salt precursor in the synthesis process. Both of these impurities had no influence on the reaction. Further in-depth analysis is provided by the Ce 3d core level spectra, as shown in Figure 4b. These spectra can be deconvoluted into several peaks corresponding to the Ce^3+^ and Ce^4+^ states, achieved by using Gaussian–Lorentzian function fitting. The “U” labeled peaks correspond to the 3d_3/2_ states (higher binding energy), and the peak labeled “V” corresponds to the 3d_5/2_ states (lower B.E.). Specifically, the peaks at 885.91 eV (V′) and 904.69 eV (U′) correspond to the 3d_5/2_ and 3d_3/2_ core levels in Ce^3+^. In contrast, the peaks at 882.80, 889.23, and 898.63 eV and 901.35, 907.50, and 917.07 eV are associated with the 3d_5/2_ and 3d_3/2_ levels in Ce^4+^.^36–39^ Before the electrochemical nitrate reduction reaction, the Ce^3+^ state is calculated to be approximately 42.8% of the total cerium. The analysis of the Fe 2p core level, also depicted in Figure 4c, reveals two core levels: 2p_1/2_ and 2p_3/2_. Notably, the Fe 2p_3/2_ peak can be divided into two distinct peaks through Gaussian–Lorentzian curve fitting, indicating the presence of Fe^2+^ and Fe^3+^ ions. Peaks corresponding to these ions are seen at binding energies of 710.66 and 712.41 eV, respectively.^39^ The formation of Fe^2+^ occurs during the synthesis of perovskite oxides (ABO_3_), where oxygen vacancies are commonly observed. These vacancies act as electron donors,^27^ facilitating the reduction of Fe^3+^ ions to Fe^2+^. Both Fe^2+^ and Fe^3+^ valences are present, as evidenced in the XPS, suggesting the occurrence of oxygen vacancies due to the space charge distribution. This is a crucial factor for the eNO_3_RR process, and the near-equal intensities of Fe^3+^ and Fe^2+^ peaks lead to the conclusion that about 52.9% of iron is in the Fe^2+^ valence state. Oxygen vacancies and Fe valence shift may play important roles for the eNO_3_RR. The O 1s spectra (Figure 4d) can be well fitted into three peaks at 529.91, 531.09, and 532.64 eV corresponding to the oxygen lattice (O_L_), oxygen vacancies (O_V_s), and adsorbed oxygen (O_ads_) identified above and supported in previous reported reference.^32,39–41^ Thus, combined XRD, Raman, XPS, and EDS analysis evidence the synthesis of CeFeO_3_/CeO_2_ composite catalyst.

**Figure 4:**
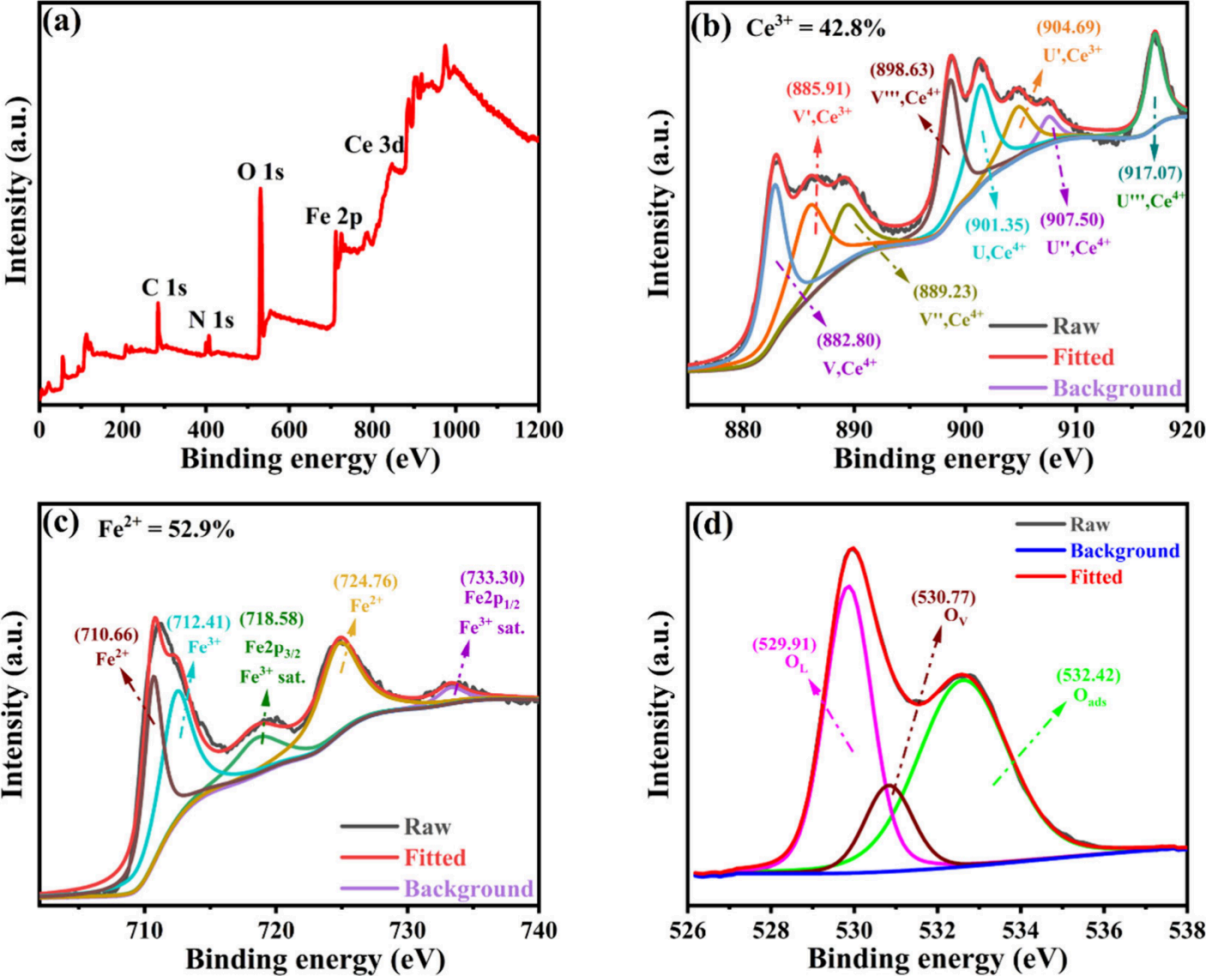
X-ray photoelectron spectroscopy spectra of CeFeO_3_/CeO_2_ composite (a) survey spectrum; deconvoluted spectra of (b) Ce 3d, (c) Fe 2p, and (d) O 1s.

### 3.2 Electrochemical Nitrate Reduction Reaction Performance of CeFeO_3_/CeO_2_ Composite Catalysts

Prior to the electrochemical nitrate reduction reaction (eNO_3_RR), linear sweep voltammetry analysis of the CeO_2_ electrode and CeFeO_3_ supported CeO_2_ electrode was performed in an argon-saturated 0.1 M KOH solution, ranging from 0.3 to −0.7 V vs RHE at a scan rate of 5 mV s^–1^, as depicted in Figure 5a. The addition of CeFeO_3_ to a CeO_2_ support suppresses HER by increasing the onset potential from −0.25 to −0.35 V seen from Figure S9b. The presence of Fe in two different oxidation states is again confirmed by the redox peak of Fe^2+^ to Fe^3+^ at 0.34 and −0.06 V in cyclic voltammetry shown in Figure S9a. To measure the charge transfer resistances during the eNO_3_RR, electrochemical impedance spectroscopy (EIS) was conducted at −0.45 V (Figure 5b) and tabulated in Tables 1 and S3. The EIS plots (Figure S9c) for both CeO_2_ and CeFeO_3_/CeO_2_ catalysts at open circuit potential (0.07 V) exhibit a single semicircle, indicative of plausible intrinsic charge transfer resistance (*R*_Intrinsic_ or *R*_I_) within the catalyst system. The measured *R*_I_ values are 2.35 Ω·cm^2^ for CeO_2_ and 1.61 Ω·cm^2^ for CeFeO_3_ supported CeO_2_, respectively. Since no other electrochemical reactions occur under these potentials, the semicircle likely represents the intrinsic charge transfer process within the catalyst. Under eNO_3_RR conditions (at −0.45 V vs RHE), as shown in Table S3, *R*_I_ values exhibit a nearly linear decrease with increasing Fe content in CeFeO_3_/CeO_2_ (Figure S10f). This trend strongly suggests that the first semicircle corresponds to intrinsic charge transfer (*R*_I_) within the catalyst system, while the second semicircle is associated with nitrate reduction (*R*_CT_). For comparison, the introduction of CeFeO_3_ (50% Fe) on CeO_2_ support significantly reduces the intrinsic charge transfer resistance (*R*_I_) from 2.23 Ω·cm^2^ for CeO_2_ to 1.51 Ω·cm^2^ for the CeFeO_3_/CeO_2_ composite, demonstrating the effect of lower internal charge transfer resistance which may cause the enhancement of the catalytic NO_3_RR kinetic charge transfer seen in lower *R*_CT_ of CeFeO_3_. Furthermore, the charge transfer resistance for nitrate reduction (*R*_CT_) decreases by 1.45 Ω·cm^2^, from 8.94 Ω·cm^2^ in pure CeO_2_ to 7.49 Ω·cm^2^ in the CeFeO_3_/CeO_2_ composite. These reductions confirm the impact of the CeFeO_3_ addition on facilitating charge transfer within the catalyst, thereby enhancing the overall performance of the system.

**Table 1:** Resistance Values of CeO_2_ and CeFeO_3_/CeO_2_ Catalysts Obtained from EIS Measurements at −0.45 V in 0.1 M KOH with 0.1 M KNO_3_

sample	*R*_s_, Ω·cm^**2**^	*R*_Intrinsic_, Ω·cm^**2**^	*R*_CT_, Ω·cm^**2**^
CeO_2_	3.47	2.23	8.94
CeFeO_3_/CeO_2_	3.43	1.51	7.49

**Figure 5:**
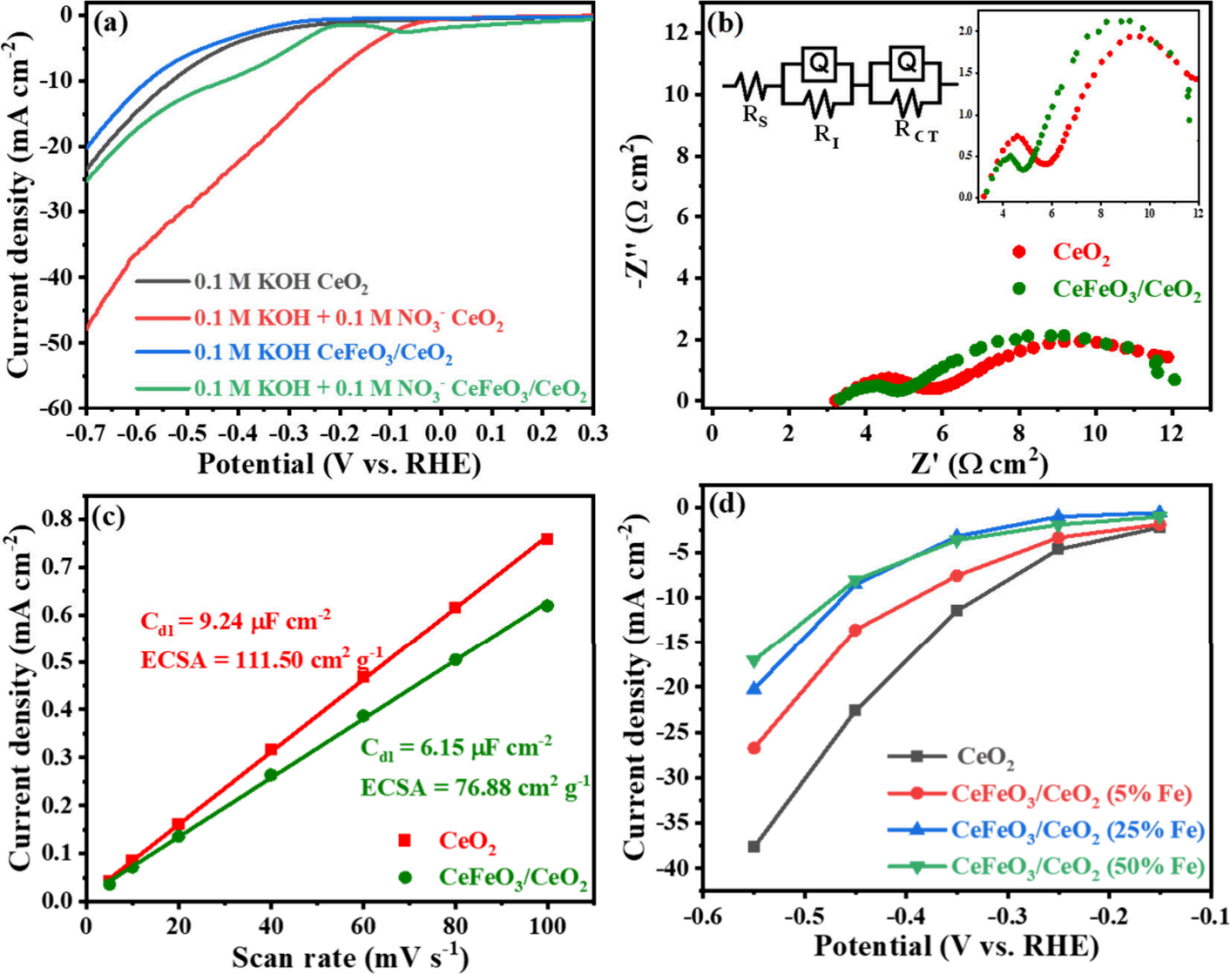
(a) Linear sweep voltammogram (5 mV s^–1^), (b) electrochemical impedance spectroscopy recorded at −0.45 V, (c) *C*_dl_ values of CeO_2_ and CeFeO_3_/CeO_2_ in Ar saturated 0.1 M KOH with 0.1 M NO_3_^–^, and (d) partial current density of nitrate reduction at different potentials.

The electrochemical accessible surface area (ECSA) of CeO_2_ and CeFeO_3_-modified CeO_2_ was calculated from the double layer capacitance (*C*_dl_), as shown in Figures 5c and S10. Increasing the CeFeO_3_ content in CeO_2_ reduces the ECSA, from 111.50 cm^2^ g^–1^ for CeO_2_ to 76.88 cm^2^ g^–1^ for CeFeO_3_ supported CeO_2_ (Table S4). This trend is reflected in the current densities, which decrease with higher Fe addition compared to CeO_2_ alone (Figure 5d). Although this might be expected to negatively impact the catalyst’s electrochemical performance, the primary current contribution for CeO_2_ is due to HER (Figure 5a).

Chronoamperometric electrolysis (CA) was conducted on various catalyst systems for 1 h at seven selected potentials ranging from 0.05 to −0.55 V in an argon-saturated 0.1 M KOH solution containing 0.1 M NO_3_^–^ (Figure S11). After each CA run, the products were quantified (Figure S12). The ammonia production rates and corresponding Faradaic efficiencies (FE) were charted in Figure 6. The CeO_2_ support (Figures 6a and 6c) exhibited a linear increase in the ammonia yield rate, from 288.8 ± 20.8 μg h^–1^ cm^–2^ at −0.05 V to 4040.4 ± 262.5 μg h^–1^ cm^–2^ at −0.45 V, with FE decreasing to 52.8 ± 2.8%, indicating significant parasitic reactions, mainly HER (Figure S13). Introducing CeFeO_3_ on CeO_2_ slightly lowered the ammonia yield rate by up to 11.5% at −0.55 V but improved the FE to 30.5% (Figures 6b and 6d). The decrease in ammonia yield rate at −0.45 V, shown in Figure 6e, with increasing Fe content, along with the rise in FE, suggests CeFeO_3_ enhances selectivity toward ammonia rather than HER. The two-electron reduction of nitrate to nitrite also increased from 629.3 ± 13.2 to 1977.9 ± 35.6 μg h^–1^ cm^–2^ with more CeFeO_3_ on CeO_2_. For hydroxylamine, 5% Fe addition reduced the yield rate, but further CeFeO_3_ formation increased it. Overall, the increased CeFeO_3_ support on CeO_2_ enhances eNO_3_RR performance, raising the overall FE to 95.1 ± 3.3%, with contributions of 6.7 ± 0.9% for nitrite, 8.3 ± 1.2% for hydroxylamine, and only 4.9 ± 0.9% loss to HER. A secondary validation of ammonia quantification was performed using the ammonia ion-selective electrode method, as presented in Table S5. The electrolyte was analyzed in its original state, while the trap solution was adjusted to pH 13 by adding 2 M KOH. The measured ammonia concentrations were then compared with those obtained using the Indophenol Blue method. The average deviations between the two methods were 5.0% for the electrolyte samples and 6.0% for the trap samples.

**Figure 6:**
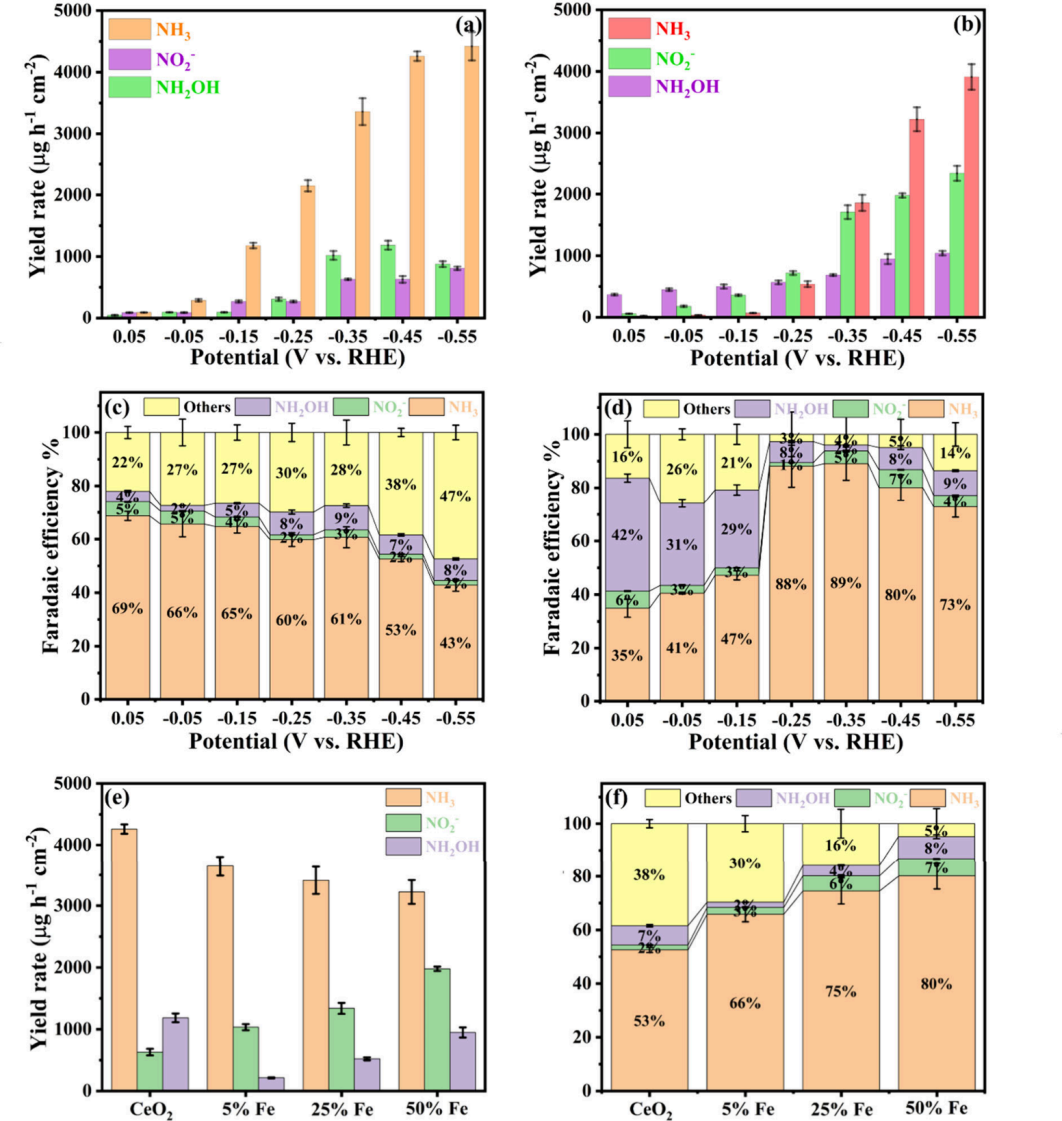
Yield rate of (a) CeO_2_ and (b) CeFeO_3_/CeO_2_; Faradaic efficiency distribution of (c) CeO_2_ and (d) CeFeO_3_/CeO_2_ at selected applied potentials in Ar saturated 0.1 M KOH with 0.1 M NO_3_^–^; (e) yield rate comparison of different Fe contents; and (f) respective FE distribution at −0.45 V vs RHE in Ar saturated 0.1 M KOH with 0.1 M NO_3_^–^.

The superior catalytic performance of the CeFeO_3_ composite is largely due to the intrinsic defects within its perovskite structure, especially oxygen vacancies. In the ABO_3_-type perovskite oxides, such as CeFeO_3_, these oxygen vacancies are not mere imperfections but act as critical enablers of enhanced catalytic activity.^42^ The mixed valency of iron where Fe^2+^ and Fe^3+^ states coexist leads to the formation of these vacancies, as the material compensates for charge imbalances.^10^ Thus, these vacancies effectively serve as active catalytic sites that facilitate various electron transfer reactions.^27,40^ In the context of nitrate reduction to ammonia, oxygen vacancies provide sites for adsorption and activation of nitrate ions,^6,43^ thereby lowering activation energies and enabling improved kinetics for the conversion process. The formation of new adsorption sites after CeFeO_3_ was supported on CeO_2_, as confirmed by N_2_ temperature-programmed desorption (TPD) profiles (Figure 7c). CeO_2_ exhibited a broad adsorption peak from 180 to 300 °C, indicating N_2_ physisorption. XRD data post-N_2_-TPD (Figure S14a) confirmed the stability of the CeO_2_ phase. CeFeO_3_ supported CeO_2_ showed strong N_2_ chemisorption at 499 and 533 °C, with additional physisorption at 200–300 °C. The higher adsorption temperatures suggest strong N_2_ adsorption due to oxygen vacancies. Online mass spectra with N_2_-TPD (Figure S15) revealed N_2_O desorption at 499 and 533 °C, confirming the involvement of lattice oxygen from CeFeO_3_ supported CeO_2_. The temperature-programmed reduction (H_2_-TPR) analysis of CeFeO_3_/CeO_2_, as shown in Figures 7a and 7b, exhibits signals signifying the decomposition of the CeFeO_3_ phase starting at 450 °C. The initial reduction occurs between 442 and 545 °C, corresponding to the decomposition of CeFeO_3_ into CeO_2_ and Fe_2_O_3_, which corroborates by previous literature.^44,45^ This is followed by sequential reductions of Fe_2_O_3_ to Fe_3_O_4_ (546–620 °C), Fe_3_O_4_ to FeO (620–678 °C), and finally, FeO to metallic Fe (678–728 °C).^44,45^ The H_2_ consumption associated with each reduction step is calculated from the area of the curve and summarized in Table S6. The total H_2_ uptake required for the complete reduction of CeFeO_3_ to CeO_2_ and metallic Fe was calculated to be 4.57 mmol g^–1^ from the TPR, which is slightly lower than the theoretical value of 4.83 mmol g^–1^,^44^ expressing a near-complete reduction. Additionally, the peak observed between 300 and 400 °C corresponds to the surface reduction of CeO_2_,^46^ which is also evident in pure CeO_2_ (Figure S16a). XRD data post-H_2_-TPR confirmed the dissociation of the CeFeO_3_ phase (see Figure S17) into CeO_2_ and metallic Fe, thereby validating the complete decomposition. The eNO_3_RR activity after TPR studies, detailed in Table S7, showed that the ammonia yield rate and FE decreases by 37% and 26% respectively, aligning with the FE of CeO_2_ alone. This loss of activity post-TPR treatment strongly correlating with structural features associated with oxygen vacancies,^3,6^ particularly in the CeFeO_3_ lattice, are essential for the enhanced eNO_3_RR performance. This further indicates a synergistic effect of CeFeO_3_ on CeO_2_ in improving the ammonia production.

**Figure 7:**
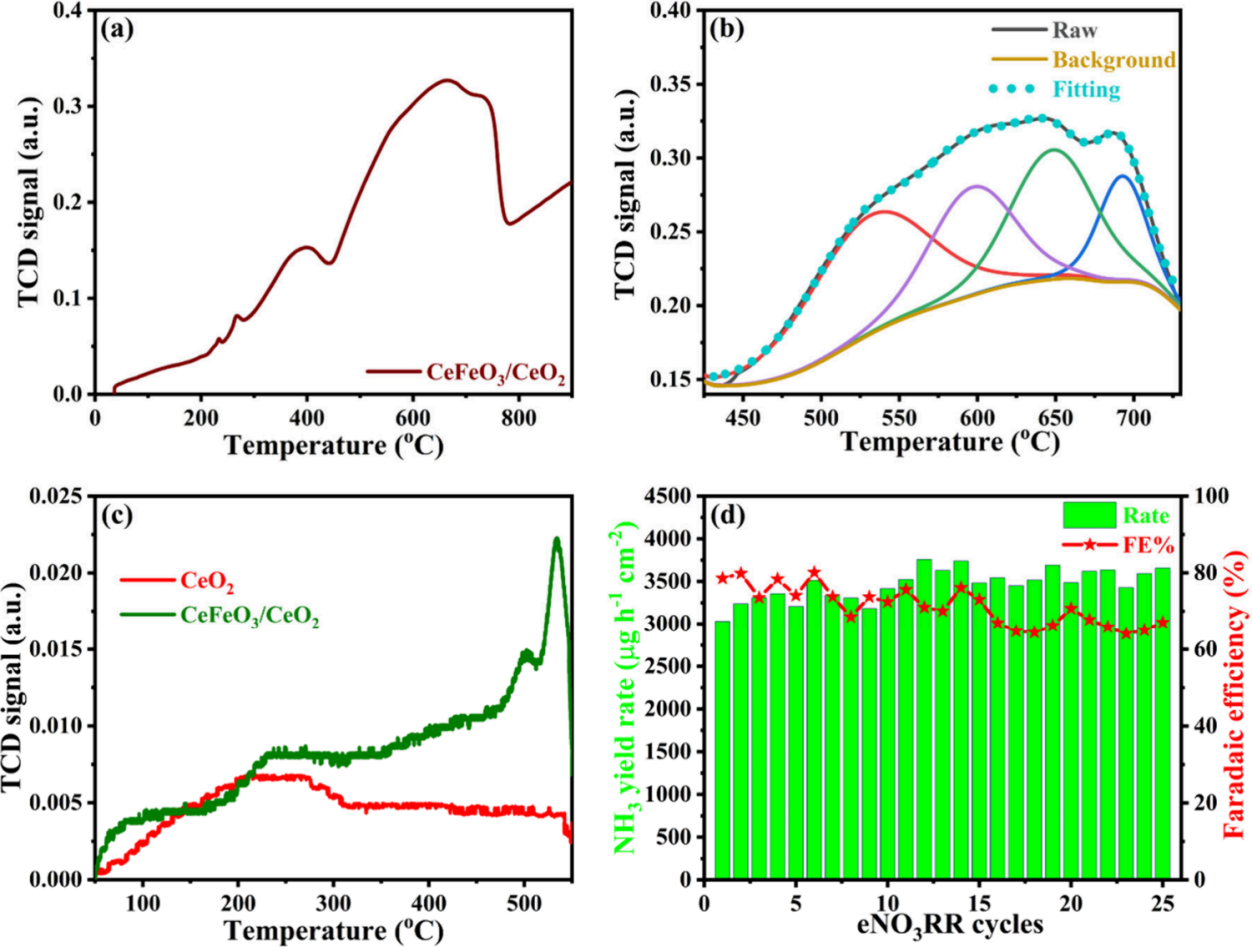
(a) H_2_ temperature programmed reduction data of CeFeO_3_/CeO_2_ and (b) deconvoluted profile of the same. (c) N_2_ temperature programmed desorption profiles of CeO_2_ and CeFeO_3_/CeO_2_ and (d) ammonia yield rate with Faradaic efficiency at 25 cycles of eNO_3_RR using a CeFeO_3_/CeO_2_ composite catalyst at −0.45 V in Ar saturated 0.1 M KOH with 0.1 M NO_3_^–^ solution.

To better understand the role of Fe in the catalytic performance, a control sample containing only iron oxide was synthesized using the same synthesis protocol but without incorporating cerium. The resulting Fe-based catalyst comprising primarily Fe_2_O_3_ and Fe_3_O_4_ (Figure S18a) phases exhibited a significantly lower ammonia yield rate of 1149.5 μg h^–1^ cm^–2^ with a Faradaic efficiency of 62.8% at −0.45 V vs RHE (Figures S18c and S18d). This performance is substantially inferior compared to that of CeO_2_ (4040.5 μg h^–1^ cm^–2^) and the CeFeO_3_/CeO_2_ composite (3223.9 ± 168.3 μg h^–1^ cm^–2^, 80.1 ± 3.3% FE), underscoring the critical role of the Ce component and the synergistic effect within the CeFeO_3_/CeO_2_ heterostructure. The poor activity of the Fe-only catalyst can be attributed to the limited nitrate/nitrogen adsorption (Figure S18b) and slower electron transfer kinetics typically associated with Fe_2_O_3_ and Fe_3_O_4_ phases.^47^

### 3.3 Stability of the CeFeO_3_/CeO_2_ Composite Catalyst

The stability of a CeFeO_3_/CeO_2_ composite catalyst was assessed at −0.45 V in an argon-saturated 0.1 M KOH solution containing 0.1 M NO_3_^–^. Chronoamperometry was performed for 1 h across 25 cycles, with electrolyte replacement in each cycle to evade the change in nitrate concentration. The yield rates of ammonia, depicted in Figure 7d, yielded 3055.5 μg h^–1^ cm^–2^ with 78.5% FE in first cycle, slowly increased in the upcoming cycles by 500 μg h^–1^ cm^–2^, and were stable for up to 25 cycles of eNO_3_RR. The ammonia FE was decreased to 65.5% after 15 cycles from 78.5% (1st cycle) and continued decreasing up to 25 cycles. The nitrite yield and FE are almost stable as ∼3416 μg h^–1^ cm^–2^ with 7.4% FE throughout the cycles (Figure S19a). Interestingly, the hydroxylamine rate was 426.8 μg h^–1^ cm^–2^ with 5.4% FE in the initial 15 cycles, then increases drastically to 1354.5 μg h^–1^ cm^–2^ with 12.5% FE from 16th cycles as illustrated in Figure S19b.

This increase in the hydroxylamine can be attributed to the phase change of Fe^3+^/Fe^2+^ atoms into Fe^0^ in the composite catalyst. This can be well visible in Figure S20a, which represents the XRD data of CeFeO_3_/CeO_2_ composite coated electrode before and after 25 cycles of eNO_3_RR. The electrode after 25 eNO_3_RR cycles expressed the formation of an Fe metallic phase with characteristic peaks at 44.6°, 65.0°, and 82.3° 2θ values representing the (110), (200), and (211) planes of cubic iron (JCPDS 01-087-0721). This Fe phase change highly affected the product selectivity of ammonia toward hydroxylamine by ∼10% without a change in nitrite. The same phase change of Fe was observed in the physical mixing of the Fe_2_O_3_/CeO_2_ catalyst (Figure S20b), and it is evident that the reduced Fe is from the minor Fe_2_O_3_ present in the system. This change did not affect the ammonia formation rate as it resulted from the CeFeO_3_ phase supported on CeO_2_. To further validate the chemical surface changes, postelectrochemical cycling XPS analysis was conducted on the CeFeO_3_/CeO_2_ electrode, as described in the experimental section of the Supporting Information, and the results are presented in Figure 8 (survey spectra in Figure S21). For comparison, another electrode was also analyzed after HER testing in the presence of KOH control experiments (NO_3_^–^ free solution).

**Figure 8:**
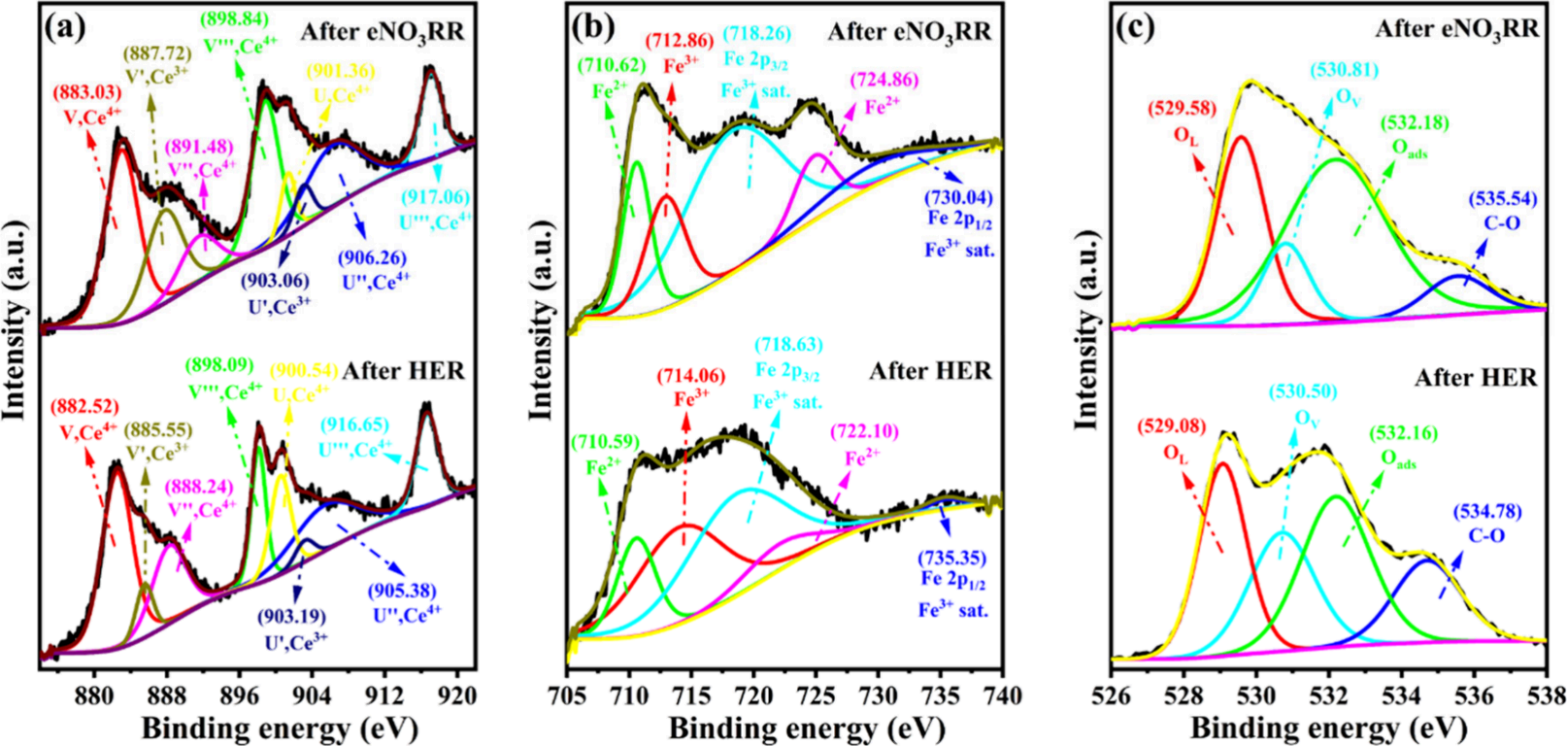
XPS deconvoluted spectra of (a) Ce 3d, (b) Fe 2p, and (c) O 1s of the CeFeO_3_/CeO_2_ composite after 25 h of HER and eNO_3_RR at −0.45 V.

The deconvoluted Ce 3d spectra of the CeFeO_3_/CeO_2_ electrodes after the HER and eNO_3_RR are compared in Figure 8a. Initially, the pristine sample (before HER and eNO_3_RR) exhibited 42.8% Ce^3+^ in the overall composition. This percentage decreased to 18.2% after HER in the absence of nitrate and slightly reduced to 37.2% Ce^3+^ following eNO_3_RR (Table S8), where different electrodes were used for each process. Additionally, a slight shift in the peaks was observed after the eNO_3_RR. For Fe^2+^, the pristine sample contained 52.3%, which reduced to 23.3% after the HER and slightly increased to 25.7% following the eNO_3_RR using separate electrodes. The O 1s spectra showed minimal compositional changes after eNO_3_RR, except for the emergence of a new C–O peak at 534.54 eV,^6^ attributed to the binder added during catalyst coating. After HER, oxygen vacancies increased by 14.4%, corresponding to a lower concentration of Ce^3+^ along with 23.3% Fe^2+^, indicating unutilized vacancies. In the case of nitrate reduction, these vacancies were maintained for 25 h of constant potential amperometry (CA) and electrolyte replacement cycles, suggesting intrinsic charge transfer between Ce and Fe, which preserved these vacancies for fresh nitrate adsorption. Although metallic iron was detected in the XRD analysis after stability testing, it was not observed in the XPS analysis, which is attributed to the immediate oxidation of metallic iron to iron oxide.

Furthermore, ICP-OES analysis of the electrolyte showed no Fe leaching, reinforcing the compositional stability of the catalyst. Additionally, the morphological stability of the catalyst-coated electrode before and after 25 h of eNO_3_RR was analyzed by SEM. As presented in Figures S22 and S23, the sponge-like morphology characteristic of the CeFeO_3_/CeO_2_ composite was well preserved after 25 h of eNO_3_RR. EDS elemental mapping confirmed the uniform distribution of Ce, Fe, and O, with no signs of agglomeration. A moderate increase in Fe content (from 13.2% to 20.3%) and a slight decrease in O content (from 24.1% to 21.9%) were observed, likely due to the partial reduction of free Fe_2_O_3_ to metallic Fe during electrolysis. The presence of F, K, and C was also detected in EDS due to the Toray carbon electrode and binder added during electrode preparation. These findings collectively confirm that the catalyst maintains both its structural and its morphological integrity under prolonged electrochemical conditions.

A separate long-term stability test conducted for 45 h at −0.45 V in the same solution (Figure S19d) showed that ammonia concentration increased and plateaued after 24 h, likely due to diminishing nitrate concentration. Figure S19d illustrates the relationship between the current and product concentration over various durations of eNO_3_RR, highlighting a decrease in current corresponding to reduced NO_3_^–^ ion concentration. These stability tests indicate that the CeFeO_3_/CeO_2_ composite maintains stable performance for up to 25 h in eNO_3_RR cycles. Online mass spectroscopy, shown in Figure S13, confirmed the absence of gases such as N_2_O, NO, and N_2_, with only H_2_ being evolved during the reaction.

### 3.4 Kinetic and Fuel Cell Studies on CeFeO_3_/CeO_2_ Composite Catalyst

The effect of the nitrate concentration in 0.1 M KOH was studied at −0.45 V using a CeFeO_3_ supported CeO_2_ catalyst. The ammonia yield rate increased linearly from 911.1 μg h^–1^ cm^–2^ at 10 mM nitrate to 5990.5 μg h^–1^ cm^–2^ at 200 mM, with only a marginal rise at 500 mM (Figure 9a). Similarly, the FE increased linearly up to 80.1% at 100 mM before leveling off. For nitrite, the yield rate continued to rise linearly up to 500 mM, with a nearly linear trend also observed for hydroxylamine (see Figure S24). The FE distribution revealed that at lower nitrate concentrations, hydroxylamine constituted 28.4% of the products but decreased as ammonia and nitrite FE increased with higher nitrate concentrations.

**Figure 9:**
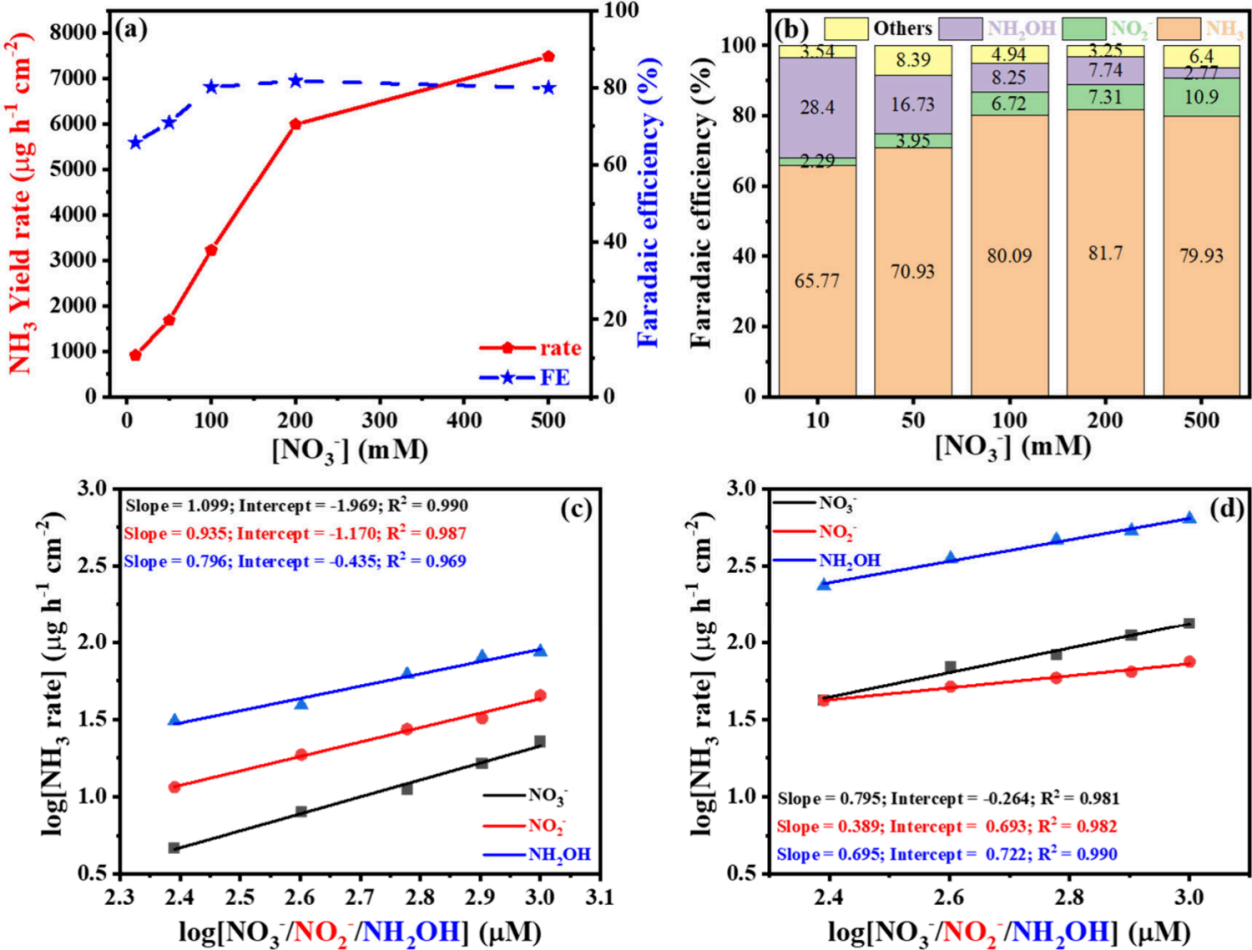
(a) Ammonia yield rate at different nitrate concentrations in 0.1 M KOH, (b) FE distribution at different nitrate concentrations, (c) reaction rate of NH_3_ on CeO_2_, and (d) reaction rate of NH_3_ on CeFeO_3_ supported CeO_2_ at −0.45 V vs RHE.

Upon support of CeFeO_3_ on CeO_2_, the nitrite yield rate increased by a factor of 3.14 compared to CeO_2_ alone, as shown in Figure 6e (the pure phase of CeFeO_3_ has not been successfully synthesized). This enhancement was accompanied by a significant improvement in the kinetics of the two-electron reduction of nitrate to nitrite. To gain deeper insight into the reaction mechanism, the initial rate constants for each reaction step (outlined in eqs 6 to 8) were determined by systematically varying the concentrations of NO_3_^–^, NO_2_^–^, and NH_2_OH from 200 to 1000 μM in 0.1 M KOH electrolyte at a potential of −0.45 V.

6 $${{\mathrm{NO}}_{3}}^{-}+6H_{2}O+8e^{-}\to {\mathrm{NH}}_{3}+9{\mathrm{OH}}^{-}\;\left(k_{1}\right)$$

7 $${{\mathrm{NO}}_{2}}^{-}+5H_{2}O+6e^{-}\to {\mathrm{NH}}_{3}+7{\mathrm{OH}}^{-}\;\left(k_{2}\right)$$

8 $${\mathrm{NH}}_{2}\mathrm{OH}+H_{2}O+2e^{-}\to {\mathrm{NH}}_{3}+{\mathrm{OH}}^{-}\;\left(k_{3}\right)$$

The ammonia yield rate was analyzed as a function of the initial reactant concentration methodology, as shown in Figures 9c and 9d. The slope of the plot represents the partial rate order, while the intercept corresponds to the initial rate constant. For reactions occurring on CeO_2_ alone, the partial rate orders for all three reactants were close to 1. The observed rate constants for ammonia formation from nitrate (*k*_1_), nitrite (*k*_2_), and hydroxylamine (*k*_3_) were 1.969, 1.170, and 0.435 s^–1^, respectively (Table 2). In contrast to CeO_2_, the CeFeO_3_/CeO_2_ composite exhibited significantly lower rate constants: 0.264 s^–1^ for nitrate, 0.693 s^–1^ for nitrite reduction, and a higher rate of *k*_3_ = 0.722 s^–1^ for hydroxylamine. These lower *k*_1_ and *k*_2_ values of CeFeO_3_ suggest that nitrate-to-ammonia conversion is slower on the composite than on pure CeO_2_. However, the conversion of NH_2_OH to ammonia as the last reduction step is 1.65 times faster on CeFeO_3_/CeO_2_ than on pure CeO_2_, presumably due to the synergetic interaction between Ce and Fe and the higher HER overpotentials observed in Figure 5a. This delay allows more time for adsorbed hydrogen (originating from water activation)^6^ to protonate NO_*x*_ intermediates (*NO → *NHO → *NHOH → *NH_2_OH → *NH_3_),^48,49^ leading to hydroxylamine and ammonia formation, as illustrated in Figure 10a. This mechanism is further supported by the significantly higher ammonia FE observed on CeFeO_3_/CeO_2_ (80.1%, Figure 6f) compared to that on CeO_2_ (52.8%).

**Table 2:** Observed Rate Constant and Partial Rate Order of the Relevant NO_*x*_ Reactants of Equations 6–8 Using CeO_2_ and CeFeO_3_/CeO_2_ Catalysts

	rate constants		rate order	
reaction	CeO_2_	CeFeO_3_/CeO_2_	CeO_2_	CeFeO_3_/CeO_2_
*k*_1_ (NO_3_^–^ → NH_3_)	1.969 s^–1^	0.264 s^–1^	1.099	0.795
*k*_2_ (NO_2_^–^ → NH_3_)	1.170 s^–1^	0.693 s^–1^	0.935	0.389
*k*_3_ (NH_2_OH → NH_3_)	0.435 s^–1^	0.722 s^–1^	0.796	0.695

**Figure 10:**
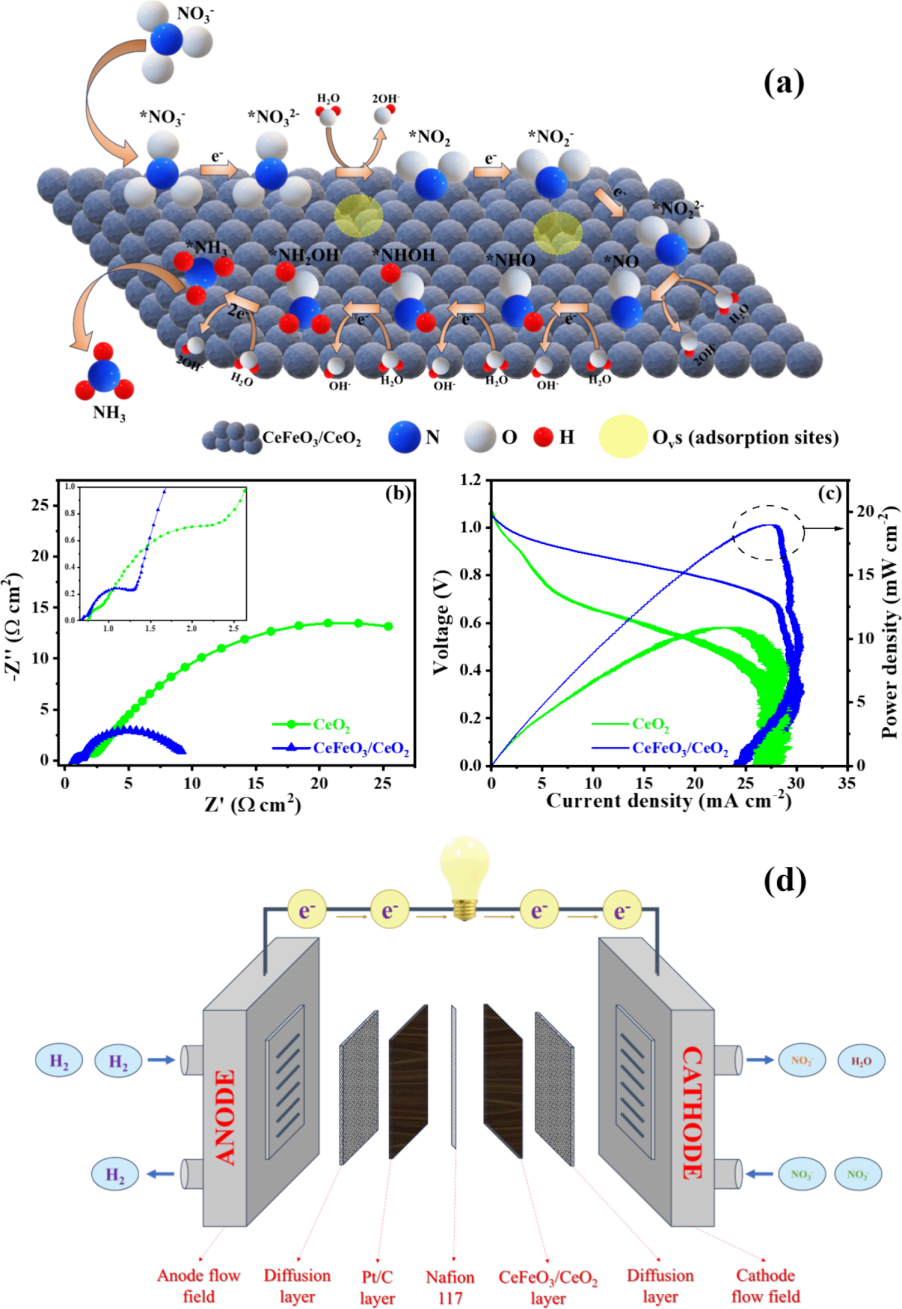
(a) Schematic illustration of nitrate reduction mechanism on CeFeO_3_/CeO_2_ matrix, (b) EIS data recorded under OCV conditions, and (c) polarization curves of H_2_–NO_3_^–^ electrochemical cells on CeO_2_ and CeFeO_3_/CeO_2_ as cathode catalysts. Measurements were taken at 35 °C with Pt/C (1.5 mg cm^–2^) as the anode and CeO_2_ (2 mg cm^–2^) or CeFeO_3_/CeO_2_ (2 mg cm^–2^) as the cathode. (d) Schematic representation of the H_2_–NO_3_^–^ fuel cell.

Additionally, the partial rate order of *k*_2_ decreases from 0.935 on CeO_2_ to 0.389 on the composite, suggesting that the reaction rate is weakly dependent on the nitrite concentration. Instead, the enhanced electron transfer synergy between CeFeO_3_ and CeO_2_ plays a key role in stabilizing adsorbed nitrate (*NO_3_^–^) and nitrite (*NO_2_^–^), allowing higher coverage of adsorbed surface NO_*x*_ in the following reduction and protonation steps leading to NH_2_OH.^46,50^ This stabilization not only promotes hydroxylamine formation but also effectively suppresses the competing HER by preventing excessive consumption of adsorbed hydrogen required for the final two electron reduction to ammonia.

Hydrogen–nitrate (H_2_–NO_3_^–^) fuel cells represent a relatively unexplored yet promising technology for sustainable green energy production, particularly in the context of the utilization of nitrate-contaminated waste. The process includes recycling the reduced nitrite (NO_2_^–^), which can be oxidized back to nitrate (NO_3_^–^) by using an oxygen source. This is feasible because the reduction potential of the NO_2_^–^/NO_3_^–^ couple is lower than that of the oxygen reduction reaction,^51^ as demonstrated in eqs 9 to 11.^51^

$${{\mathrm{NO}}_{3}}^{-}+2H^{+}+2e^{-}\to {{\mathrm{NO}}_{2}}^{-}+H_{2}O,E^{0}=0.94\;V$$ 9

$${{\mathrm{NO}}_{2}}^{-}+0.5O_{2}\to {{\mathrm{NO}}_{3}}^{-},\Delta G^{0}=-18.36\;{\mathrm{kcal mol}}^{-1}$$ 10

$${{\mathrm{NO}}_{3}}^{-}+4H^{+}+3e^{-}\to \mathrm{NO}+2H_{2}O,E^{0}=0.96\;V$$ 11

In our study, we evaluated the performance of H_2_–NO_3_^–^ fuel cells using Pt/C as the anode catalyst. The detailed operating procedure is provided in the Methods section of the Supporting Information, and the fuel cell setup is schematically illustrated in Figure 10d. The open-circuit voltage (OCV) measurements showed values of 0.907 V for CeO_2_ and 0.906 V for CeFeO_3_/CeO_2_, stable for over an hour (Figure S25a). The electrochemical impedance spectroscopy (EIS) spectra, shown in Figure 10b, indicate two distinct charge transfer resistances for each catalyst. These two resistances correspond to the reduction of NO_3_^–^ to NO_2_^–^ and the subsequent reduction to NO, as further confirmed by the online mass spectrometry of the fuel cell (Figure S25c). For the CeO_2_ catalyst, the first charge transfer resistance was 5.16 Ω·cm^2^, which decreased to 1.60 Ω·cm^2^ for CeFeO_3_/CeO_2_. The second charge transfer resistance was measured at 59.02 Ω·cm^2^ for CeO_2_ and 16.90 Ω·cm^2^ for CeFeO_3_/CeO_2_. Additionally, the intrinsic charge transfer resistances for CeO_2_ and CeFeO_3_/CeO_2_ were 1.05 and 0.44 Ω·cm^2^, respectively.

The peak power density for the CeO_2_ catalyst reached 11.4 mW cm^–2^ at 0.456 V (Figure 10c). After incorporating CeFeO_3_ on CeO_2_, the peak power density increased to 19.2 mW cm^–2^ at 0.702 V. The highest current density observed was 29.7 mA cm^–2^ at 0.466 V with CeFeO_3_ supported CeO_2_ catalyst while CeO_2_ produced a current density of 23.5 mA cm^–2^ at a similar potential. The thermodynamic efficiency of the catalysts was calculated using eq 12 as follows: 45.8% and 74.6% for CeO_2_ and CeFeO_3_/CeO_2_, respectively. Although this fuel cell could not completely reduce NO_3_^–^ to NH_3_ due to thermodynamic limitations, the electrical energy generated under galvanic conditions can be utilized to drive the subsequent electrolytic reduction of NO_2_^–^ to NH_3_. These findings demonstrate the potential of CeFeO_3_ supported systems to enable a green and environmentally friendly ammonia production process while simultaneously generating electrical power in an H_2_–NO_3_^–^ fuel cell configuration. The performance of this system is comparable to other recently reported fuel cell technologies (as detailed in Table S9) and are similar to direct methanol fuel cells.^52,53^

12 $$\mathrm{thermodynamic efficiency}\;\%=\frac{E_{out}}{E_{theoretical}}\times 100$$

## 4 Conclusion

In this study, CeFeO_3_ supported CeO_2_ catalysts were synthesized using a microwave-assisted method and comprehensively characterized by XRD, Raman, SEM, EDS, BET, HR-TEM, and XPS techniques. The characterization results indicated that 50% Fe added to CeO_2_ resulted in a composite with a predominant CeFeO_3_ phase supported on CeO_2_. Electrochemical evaluations of nitrate reduction for each catalysts demonstrated that the CeO_2_ support achieved a high ammonia yield rate of 4040.5 ± 262.5 μg h^–1^ cm^–2^, with a Faradaic efficiency of 52.8 ± 2.8% at −0.45 V vs RHE. The formation of a CeFeO_3_ phase on CeO_2_ notably enhanced the ammonia FE 80.1 ± 3.3%, alongside an appreciable yield rate of 3223.9 ± 168.3 μg h^–1^ cm^–2^. This CeFeO_3_ phase also restricts the parasitic HER to 4.9 ± 0.9%, while the remaining goes for nitrite and hydroxylamine. The standout catalytic performance of the CeFeO_3_/CeO_2_ composite is attributed to the inherent structural defects, particularly oxygen vacancies, facilitated by the mixed valency of iron ions (Fe^2+^ and Fe^3+^). Moreover, the composite catalyst displayed consistent performance across 25 h of eNO_3_RR cycles in a 0.1 M KOH solution containing 0.1 M KNO_3_. The N_2_-TPD and H_2_-TPR analysis indirectly confirms the synergetic charge transfer effect between CeO_2_ support and CeFeO_3_ in electrochemical reduction of nitrate. These findings on the cerium orthoferrite composite’s nitrate reduction capabilities offer a significant improvement in catalyst design while harnessing orthoferrite materials for sustainable ammonia synthesis through electrochemical processes and clean power generation.

## Supplementary Material



## Notes

Experimental procedure; calibration plots; EDS mapping; BET; chronoamperometry and UV–vis plots; online mass spectroscopy plots; H_2_-TPR; XRD patterns after stability, N_2_-TPD, and N_2_-TPR; and SEM images (PDF)
