# Supplementary material for: Electrochemical Nitrogen Fixation Using CeFeO3 and CeO2 for Ammonia Synthesis and Nitrate Remediation
Source: ACS Appl Mater Interfaces. 2025 Jun 12;17(25):36796–809. doi: 10.1021/acsami.5c07123 (PMC12203462; doi:10.1021/acsami.5c07123)
Supplement: Supplementary file 1 [file am5c07123_si_001.pdf]

Supporting information

Electrochemical Nitrogen Fixation Using $CeFeO_3$ and CeO_2 for Ammonia Synthesis and Nitrate Remediation

James Ebenezer,[a] Parthiban Velayudham,[a] and Alex Schechter[*,a,b]

[a] Department of Chemical Sciences, Ariel University, Ariel 40 700, Israel.

[b] Research and Development Centre for Renewable Energy, New Technology Centre, University of West Bohemia, 301 00 Pilsen, Czech Republic.

[*] Email: salex@ariel.ac.il; Tel.: +972 39371470; Fax: +972 547740254

Contents

Figure S1. (a) UV data of nitrate standard samples in 0.1 M KOH, and (b) their corresponding calibration plot.

Figure S2. (a) UV data of nitrite standard samples in 0.1 M KOH, and (b) their corresponding calibration plot.

Figure S3. (a) UV-Visible spectra of ammonia standard samples in 0.1 M KOH with Indophenol blue, and (b) their corresponding calibration plot, (c) UV-Visible data of ammonia standard samples in 1 mM H_2SO_4 with Indophenol blue, and (d) their corresponding calibration plot.

Figure S4. (a) UV-Visible spectra of hydroxylamine standard sample solutions in 0.1 M KOH with 3 mM $K_3[Fe(CN)_6]$, and (b) their corresponding calibration plot; (c) calibration plot of ammonia ion selective electrode in 0.1 M KOH solution with standard ammonia solutions (0.1, 1.0, 10.0 ppm).

Figure S5. (a) Overall, (b) partial X-ray diffraction patterns, and (c) Raman spectra of different Fe addition to CeO_2 support.

Figure S6. SEM images of (a) CeO_2, (b) $CeFeO_3/CeO_2$ (5 at.% Fe), (c) $CeFeO_3/CeO_2$ (25 at.% Fe), and (d) $CeFeO_3/CeO_2$ (50 at.% Fe).

Figure S7. (a) Scanning electron microscopy images of $CeFeO_3$ supported CeO_2 composite at 20Kx magnification, (b, c and d) EDS mapping of oxygen, iron and cerium of the same.

Figure S8. (a) BET N_2 adsorption–desorption isotherms, and (b) corresponding pore size distributions of CeO_2 and $CeFeO_3/CeO_2$.

Figure S9. (a) cyclic voltammogram (inset shows magnified CV), (b) linear sweep voltammogram; electrochemical impedance spectroscopy results of (c) CeO_2, and $CeFeO_3/CeO_2$ at OCV (inset shows magnified spectra), and (d) under applied potentials (inset shows magnified spectra).

Figure S10. Cyclic voltammogram of (a) CeO_2, (b) $CeFeO_3/CeO_2$ (5 at.% Fe), (c) $CeFeO_3/CeO_2$ (25 at.% Fe), and (d) $CeFeO_3/CeO_2$ (50 at.% Fe), corresponding (e) C_{dl} and (f) ECSA values in 0.1 M KOH containing 0.1 M KNO_3 solution (inset shows resistances at -0.45 V vs. RHE).

Figure S11. Chronoamperometry plots of (a) CeO_2, (b) $CeFeO_3/CeO_2$ (5 at.% Fe), (c) $CeFeO_3/CeO_2$ (25 at.% Fe), and (d) $CeFeO_3/CeO_2$ (50 at.% Fe) in 0.1 M KOH containing 0.1 M KNO_3 solution.

Figure S12. UV-Visible spectra of (a) ammonia in electrolyte, (b) ammonia in trap, (c) nitrite in electrolyte, and (d) hydroxylamine in electrolyte (inset shows magnified spectra) after CA on $CeFeO_3/CeO_2$ electrode in 0.1 M KOH containing 0.1 M KNO_3 solution.

Figure S13. Online mass spectra result of (a) CeO_2, and (b) $CeFeO_3/CeO_2$ electrode in 0.1 M KOH containing 0.1 M KNO_3 solution at -0.45 V.

Figure S14. X-ray diffraction patterns of (a) CeO_2, and (b) $CeFeO_3/CeO_2$ after N_2 TPD analysis (25-550 °C).

Figure S15. (a) Online mass spectrum recorded during the degassing at 200 °C, nitrogen purging at 50 °C and N_2-TPD experiment of $CeFeO_3/CeO_2$ composite, and (b) magnified view of nitrogen and nitrous oxide gas in the region of N_2 desorption.

Figure S16. H_2-TPR data of (a) CeO_2, and (b) Fe_2O_3/Fe_3O_4.

Figure S17. X-ray diffraction patterns of $CeFeO_3/CeO_2$ after H_2 TPR analysis.

Figure S18. (a) XRD pattern, and (b) N_2-TPD profile of synthesized Fe_2O_3/Fe_3O_4; (c) Yield rate, and (d) Faradaic efficiency distribution of Fe_2O_3/Fe_3O_4 at selected applied potentials in Ar saturated 0.1 M KOH with 0.1 M NO_3^-.

Figure S19. (a) nitrite, (b) hydroxylamine, (c) FE distribution of $CeFeO_3/CeO_2$ electrode in 25 hours of eNO_3RR cycles at -0.45 V, and (d) long term eNO_3RR studies of the same.

Figure S20. X-ray diffraction patterns of (a) $CeFeO_3/CeO_2$ coated electrode, and (b) Fe_2O_3/CeO_2 coated electrode after 25 hours of eNO_3RR cycles at -0.45 V.

Figure S21. XPS survey spectrum of $CeFeO_3/CeO_2$ after HER and eNO_3RR at -0.45 V for 25 hours.

Figure S22. (a-c) Scanning electron microscopy images of $CeFeO_3/CeO_2$ coated pristine electrode at different magnifications, (d-f) EDS mapping of cerium, iron and oxygen (on Figure c), and (g) EDS spectra of the same.

Figure S23. (a-c) Scanning electron microscopy images of $CeFeO_3/CeO_2$ coated electrode at different magnifications after stability of 25-hour eNO_3RR cycles at -0.45 V in 0.1 M KOH with 0.1 M KNO_3, (d-f) EDS mapping of cerium, iron and oxygen (on Figure c), and (g) EDS spectra of the same.

Figure S24. (a) Nitrite, and (b) hydroxylamine yield rate of $CeFeO_3/CeO_2$ in different nitrate concentrated electrolyte at -0.45 V.

Figure S25. (a) OCV, (b) PEIS recorded at different potentials, and (c) Online mass spectra measurements (at OCV of 1.06) of H_2-NO_3^- fuel cell using $CeFeO_3/CeO_2$ as cathode and Pt/C anode.

Table S1. Crystallize size and calculated micro strain of CeO_2 and $CeFeO_3$ supported CeO_2 catalysts.

Table S2. EDS analysis of $CeFeO_3/CeO_2$ (50 at.% Fe) at different spots.

Table S3. Resistance values of $CeFeO_3/CeO_2$ (50 at.% Fe) catalyst obtained from EIS measurements at different applied potentials in 0.1 M KOH with 0.1 M KNO_3.

Table S4. Electrochemical active surface area (ECSA) measured under non-Faradaic region of 0.85 to 1.30 V vs. RHE and EIS measurements for CeO_2 and $CeFeO_3/CeO_2$ electrodes at -0.45 V vs. RHE in 0.1 M KOH containing 0.1 M KNO_3 solution.

Table S5. Comparison of ammonia concentration analyzed by ammonia ion selective electrode and Indophenol method.

Table S6. Hydrogen reactions result of $CeFeO_3/CeO_2$ catalyst from H_2-TPR analysis.

Table S7. eNO_3RR performance comparison of $CeFeO_3/CeO_2$ after H_2-TPR studies.

Table S8. Summary of XPS results of $CeFeO_3/CeO_2$ after HER and eNO_3RR at -0.45 V for 25 hours.

Table S9. Fuel cell performance comparison of $CeFeO_3/CeO_2$ with recently reported catalysts.

Additional Methods

XPS analysis after stability

The chemical changes after stability testing were analyzed using XPS. The $CeFeO_3/CeO_2$ catalyst (without carbon) was dispersed in a 1:1 water:IPA mixture containing 35 wt.% Nafion ionomer and sonicated thoroughly. Following sonication, the catalyst was coated onto a gold (Au) electrode with a surface area of 1.2 x 1.5 cm^2, achieving a catalyst loading of approximately 3.2 mg cm^{-2}. This $CeFeO_3/CeO_2$-coated Au electrode was then employed for eNO_3RR stability studies in 0.1 M KOH with 0.1 M KNO_3 at -0.45 V for 25 hours. After the

eNO_3RR process, the electrode was air-dried at room temperature, and the $CeFeO_3/CeO_2$ catalyst was gently scraped off for subsequent XPS analysis. The same method was applied to study HER under similar conditions, using a nitrate-free 0.1 M KOH electrolyte.

H_2-NO_3^- Fuel cell measurements

The gas diffusion layer (GDL) was prepared by mixing the respective catalyst (CeO_2, or $CeFeO_3/CeO_2$) with Vulcan XC-72 carbon (60 wt.% of total catalyst) in 5 mL of isopropanol (IPA), followed by 10 minutes of sonication. Afterward, 10 wt.% Nafion was added, and the mixture was sonicated further for 30 minutes in an ice bath. The resulting catalyst ink was then brush-coated onto Teflonized carbon paper (2x2 cm^2), dried, and used as the cathode. The same procedure was followed to prepare the Pt/C anode. Catalyst loadings of 1.5 mg cm^{-2} for the anode and 2 mg cm^{-2} for the cathode were maintained. The fuel cell was assembled using a pre-treated Nafion 117 membrane as the separator. Humidified hydrogen was supplied to the anode at a flow rate of 25 mL min^{-1}, while 2 M HNO_3 was circulated at the cathode at 2 mL min^{-1} without a back pressure regulator. All measurements were conducted at room temperature (27 °C).

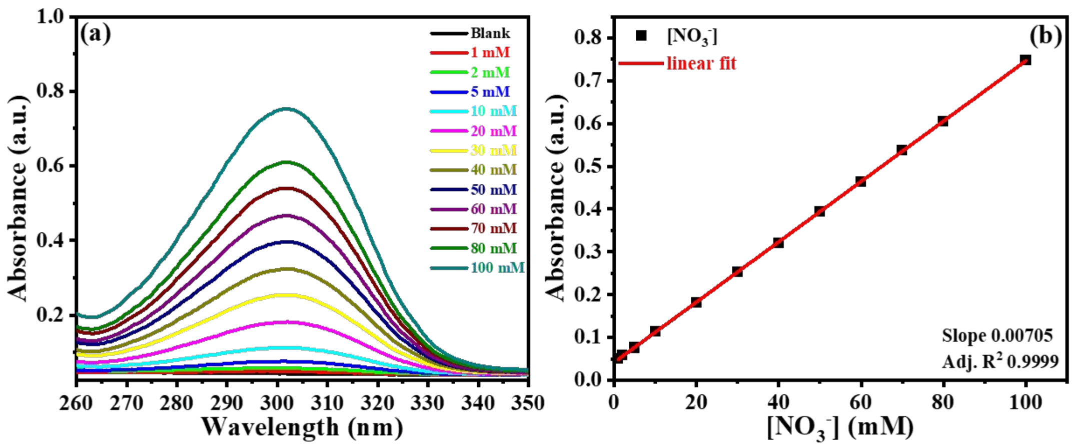

Figure S1. (a) UV data of nitrate standard samples in 0.1 M KOH, and (b) their corresponding calibration plot.

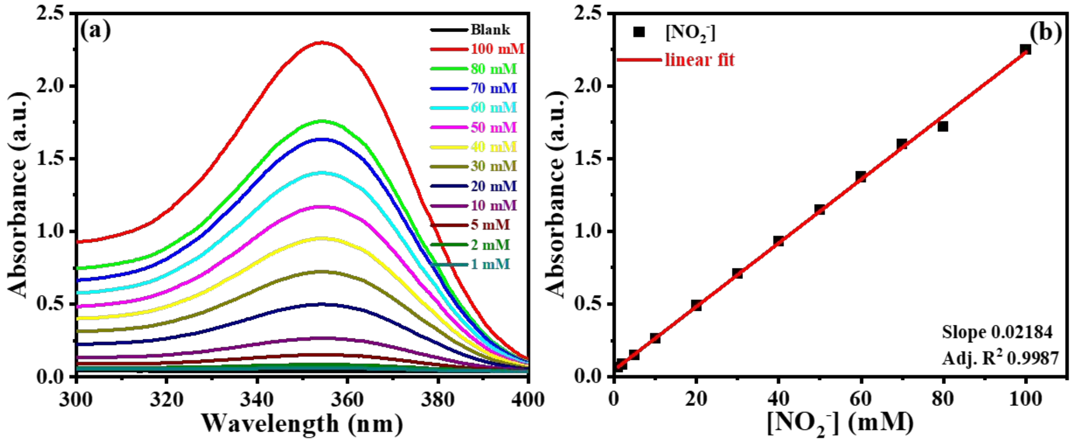

Figure S2. (a) UV data of nitrite standard samples in 0.1 M KOH, and (b) their corresponding calibration plot.

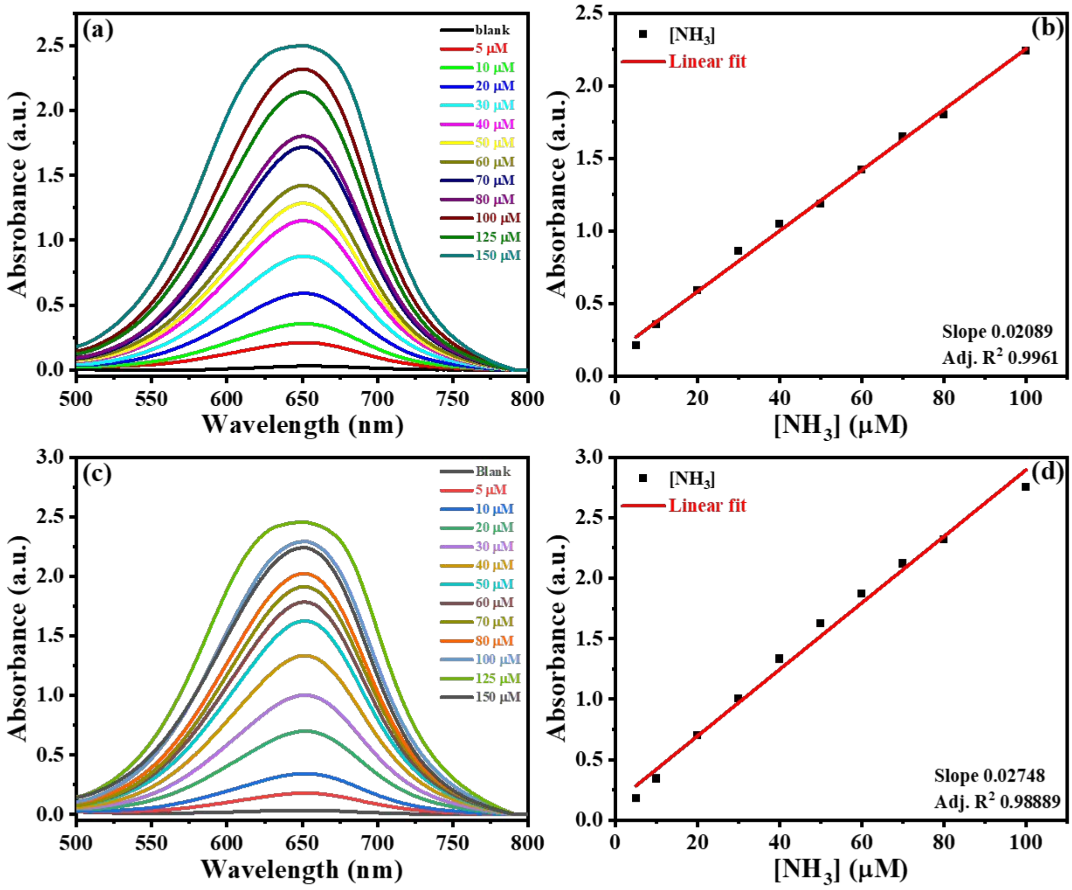

Figure S3. (a) UV-Visible spectra of ammonia standard samples in 0.1 M KOH with Indophenol blue, and (b) their corresponding calibration plot, (c) UV-Visible data of ammonia

standard samples in 1 mM H_2SO_4 with Indophenol blue, and (d) their corresponding calibration plot.

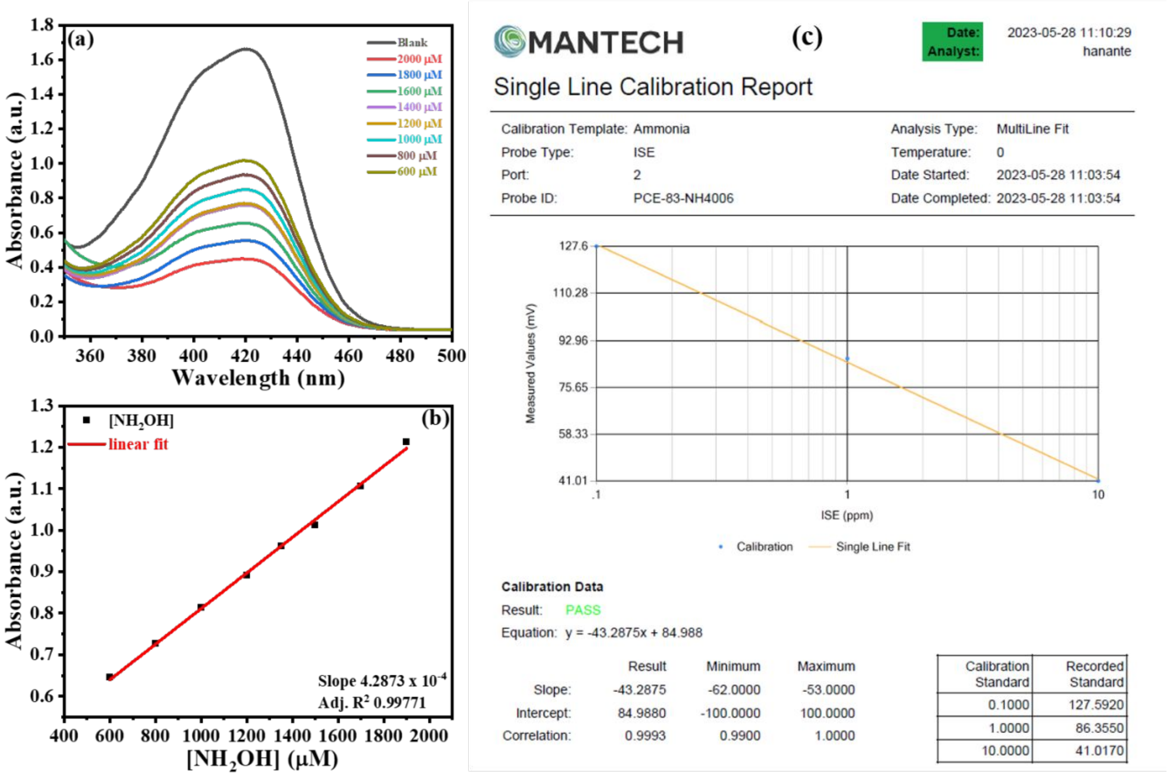

Figure S4. (a) UV-Visible spectra of hydroxylamine standard sample solutions in 0.1 M KOH with 3 mM $K_3[Fe(CN)_6]$, and (b) their corresponding calibration plot; (c) calibration plot of ammonia ion selective electrode in 0.1 M KOH solution with standard ammonia solutions (0.1, 1.0, 10.0 ppm).

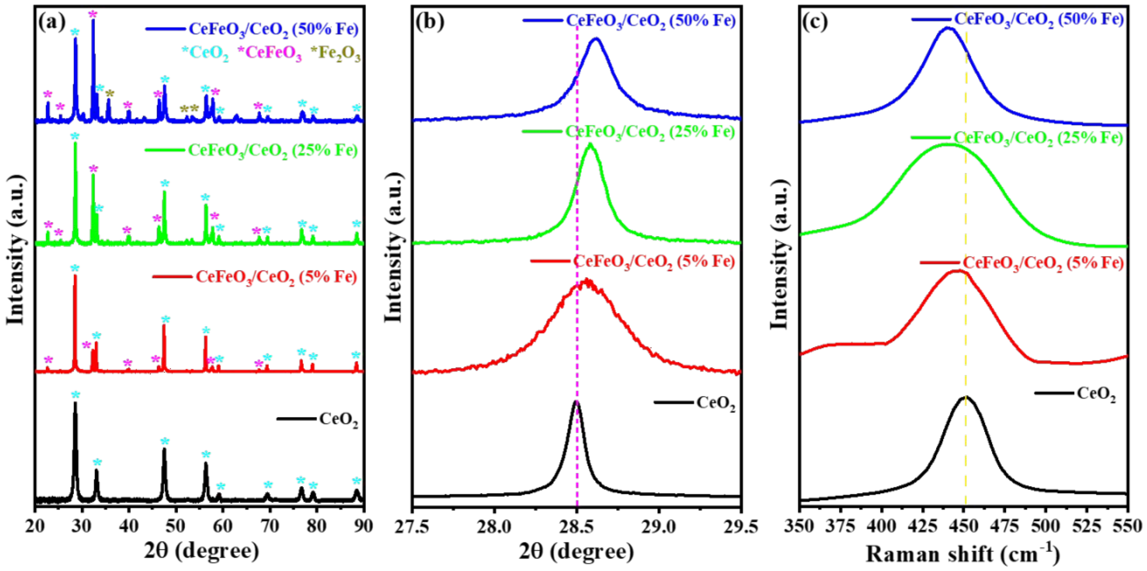

Figure S5. (a) Overall, (b) partial X-ray diffraction patterns, and (c) Raman spectra of different Fe addition to CeO_2 support.

Table S1. Crystallize size and calculated micro strain of CeO_2 and $CeFeO_3$ supported CeO_2 catalysts.

**Catalyst**	**Crystallite size (nm)**		**Peak position (2θ)**	**FWHM**	**Micro strain (ε) (x 10^3)**
	CeO_2	$CeFeO_3$			
CeO_2	20.4	-	28.49	0.433	11.65
$CeFeO_3/CeO_2$ (5% Fe)	30.0	124.8	28.52	0.098	3.01
$CeFeO_3/CeO_2$ (25% Fe)	65.7	196.4	28.58	0.192	7.31
$CeFeO_3/CeO_2$ (50% Fe)	78.3	216.8	28.62	0.197	8.47

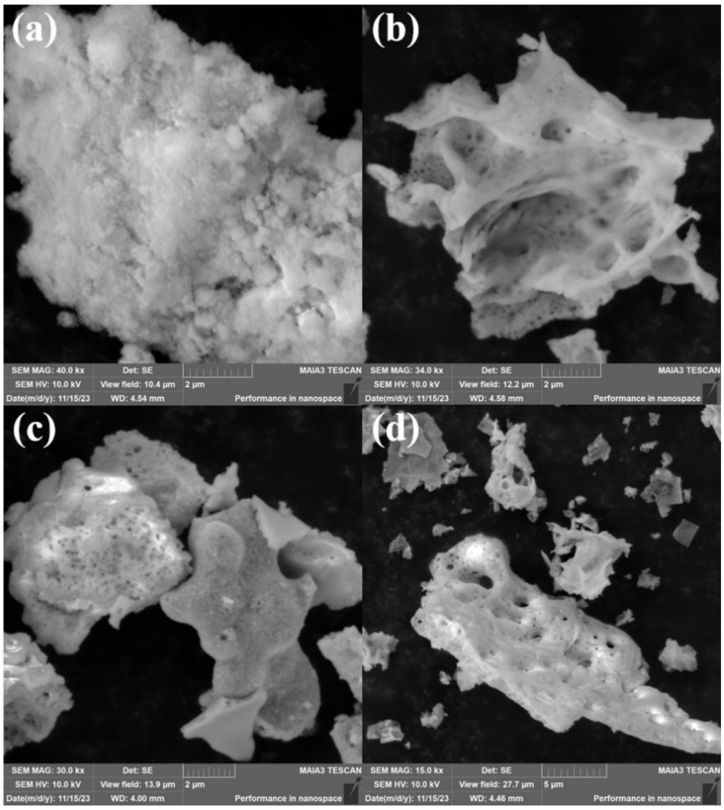

Figure S6. SEM images of (a) CeO_2, (b) $CeFeO_3/CeO_2$ (5 at.% Fe), (c) $CeFeO_3/CeO_2$ (25 at.% Fe), and (d) $CeFeO_3/CeO_2$ (50 at.% Fe).

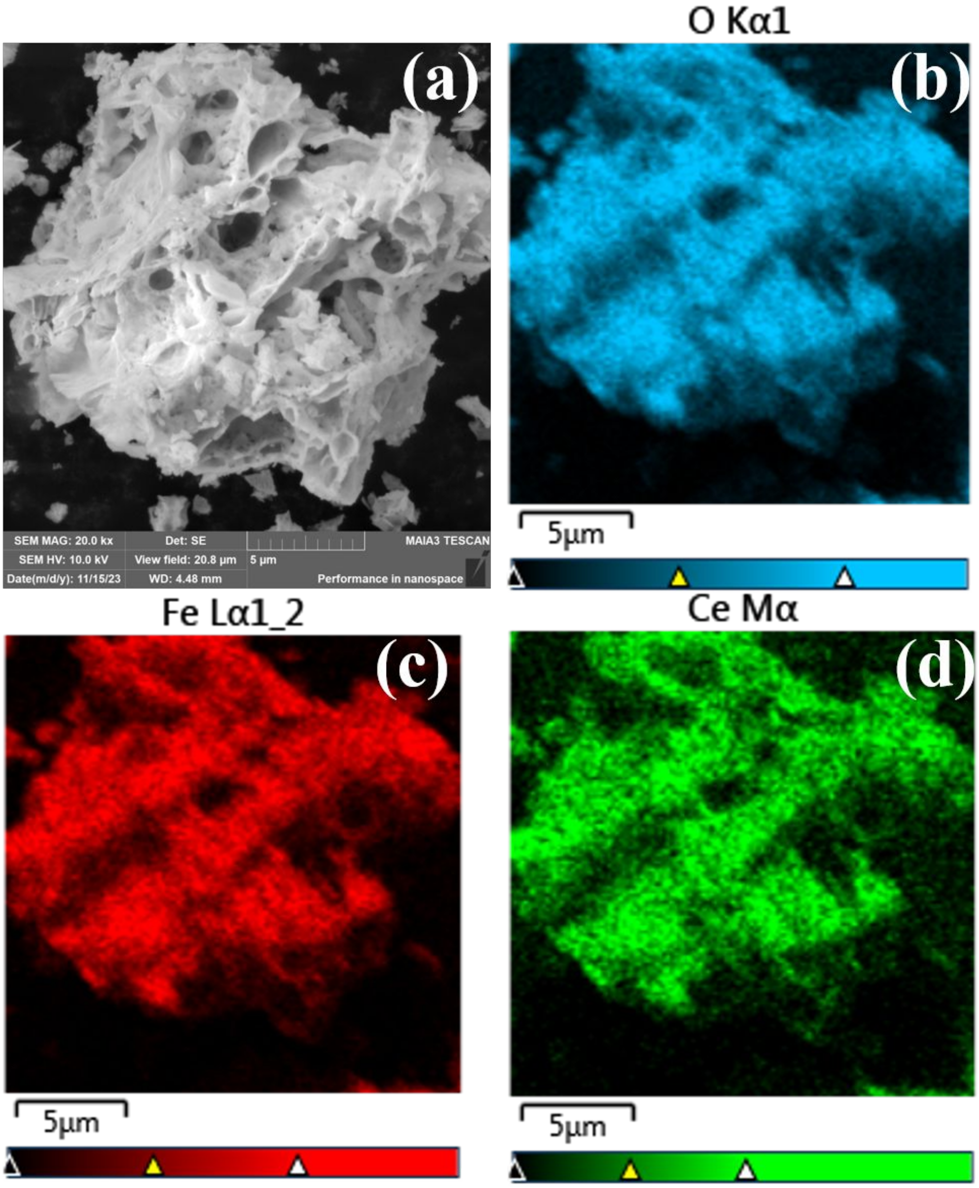

Figure S7. (a) Scanning electron microscopy images of $CeFeO_3$ supported CeO_2 composite at 20Kx magnification, (b, c and d) EDS mapping of oxygen, iron and cerium of the same.

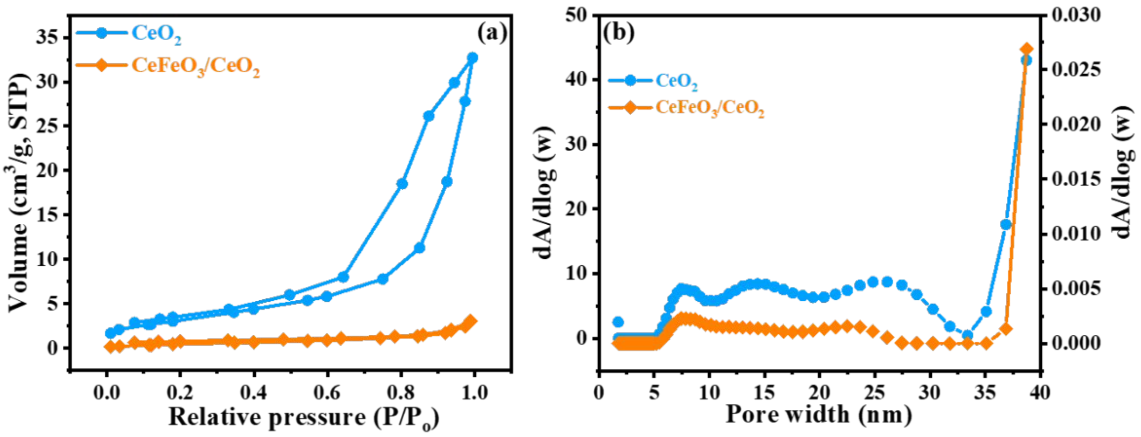

Figure S8. (a) BET N_2 adsorption-desorption isotherms, and (b) corresponding pore size distributions of CeO_2 and $CeFeO_3/CeO_2$ (50 at.% Fe).

Table S2. EDS analysis of $CeFeO_3/CeO_2$ (50 at.% Fe) at different spots.

**Elements**	**Spot 1 (Figure 3b) (Wt.%)**	**Spot 2 (Figure 3d) (Wt.%)**
Ce	76.11%	57.50%
Fe	2.11%	19.41%
O	21.78%	23.09%

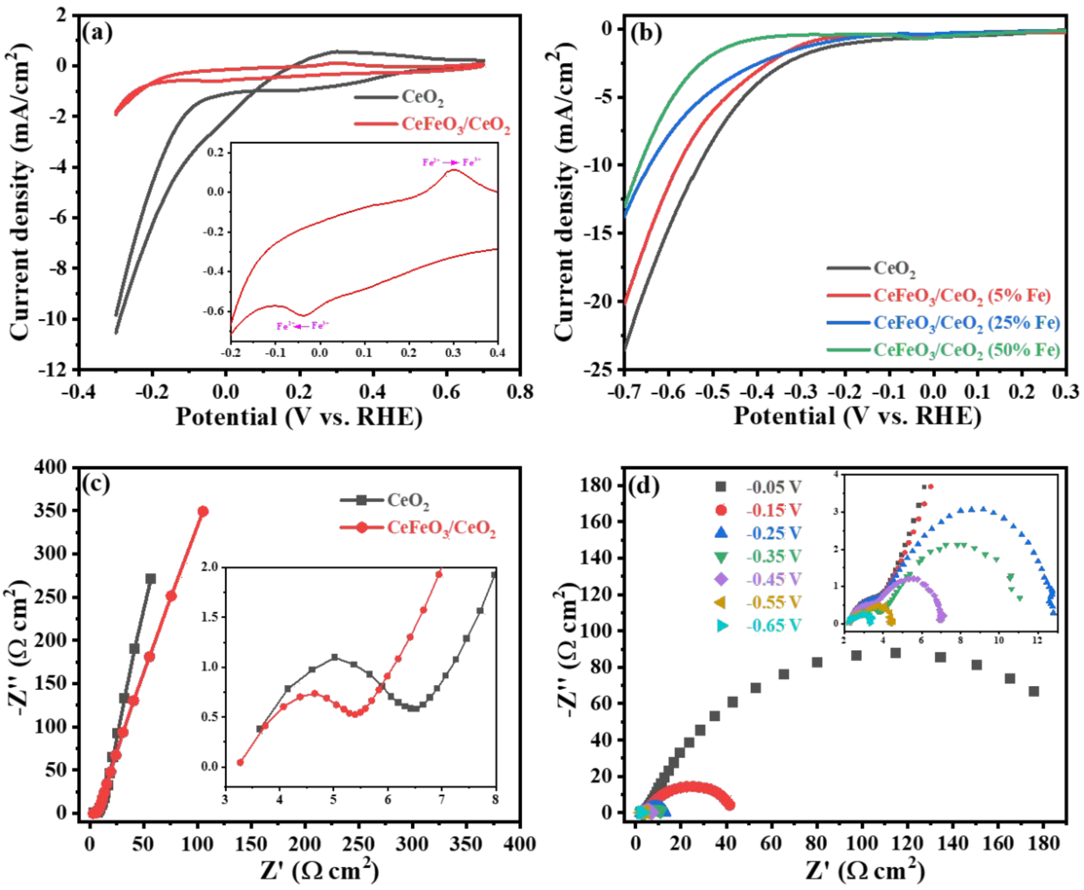

Figure S9. (a) cyclic voltammogram (inset shows magnified CV), (b) linear sweep voltammogram; electrochemical impedance spectroscopy results of (c) CeO_2, and $CeFeO_3/CeO_2$ at OCV (inset shows magnified spectra), and (d) under applied potentials (inset shows magnified spectra).

Table S3. Resistance values of $CeFeO_3/CeO_2$ catalyst obtained from EIS measurements at different applied potentials in 0.1 M KOH with 0.1 M KNO_3.

Potential (V vs. RHE)	R_s, Ω.cm^2	R_I, Ω.cm^2	R_{CT}, Ω.cm^2
-0.05	2.33	1.69	171.88
-0.15	2.31	1.67	36.89
-0.25	2.29	1.52	14.57

-0.35	2.33	1.48	8.94
-0.45	2.27	1.29	7.28
-0.55	2.27	0.66	2.90

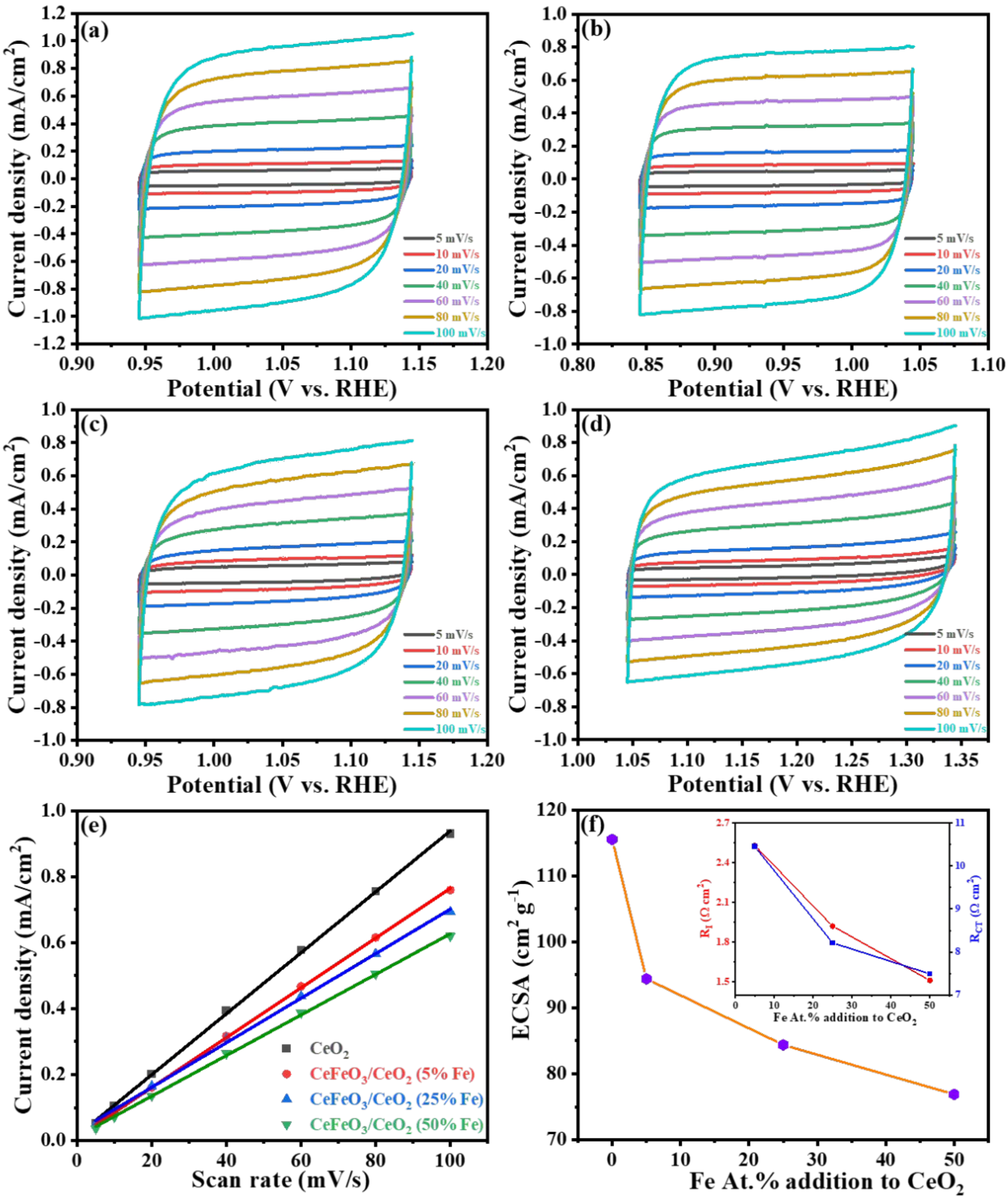

Figure S10. Cyclic voltammogram of (a) CeO_2, (b) $CeFeO_3/CeO_2$ (5 at.% Fe), (c) $CeFeO_3/CeO_2$ (25 at.% Fe), and (d) $CeFeO_3/CeO_2$ (50 at.% Fe), corresponding (e) C_{dl} and (f)

ECSA values in 0.1 M KOH containing 0.1 M KNO_3 solution (inset shows resistances at -0.45 V vs. RHE).

Table S4. Electrochemical active surface area (ECSA) measured under non-Faradaic region of 0.85 to 1.30 V vs. RHE and EIS measurements for CeO_2 and $CeFeO_3/CeO_2$ electrodes at -0.45 V in 0.1 M KOH containing 0.1 M KNO_3 solution.

**Sample**	**C_{dl} (µF cm^{-2})**	**ECSA (cm^2 g^{-1})**	**EIS measurements (Ω.cm^2)**		
			R_S (R_1)	R_I (R_2)	R_{CT} (R_3)
CeO_2	9.24	115.50	3.47	2.23	8.94
$CeFeO_3/CeO_2$ (5% Fe)	7.55	94.38	3.21	2.53	10.45
$CeFeO_3/CeO_2$ (25% Fe)	6.75	84.38	3.23	1.92	8.21
$CeFeO_3/CeO_2$ (50% Fe)	6.15	76.88	3.43	1.51	7.49

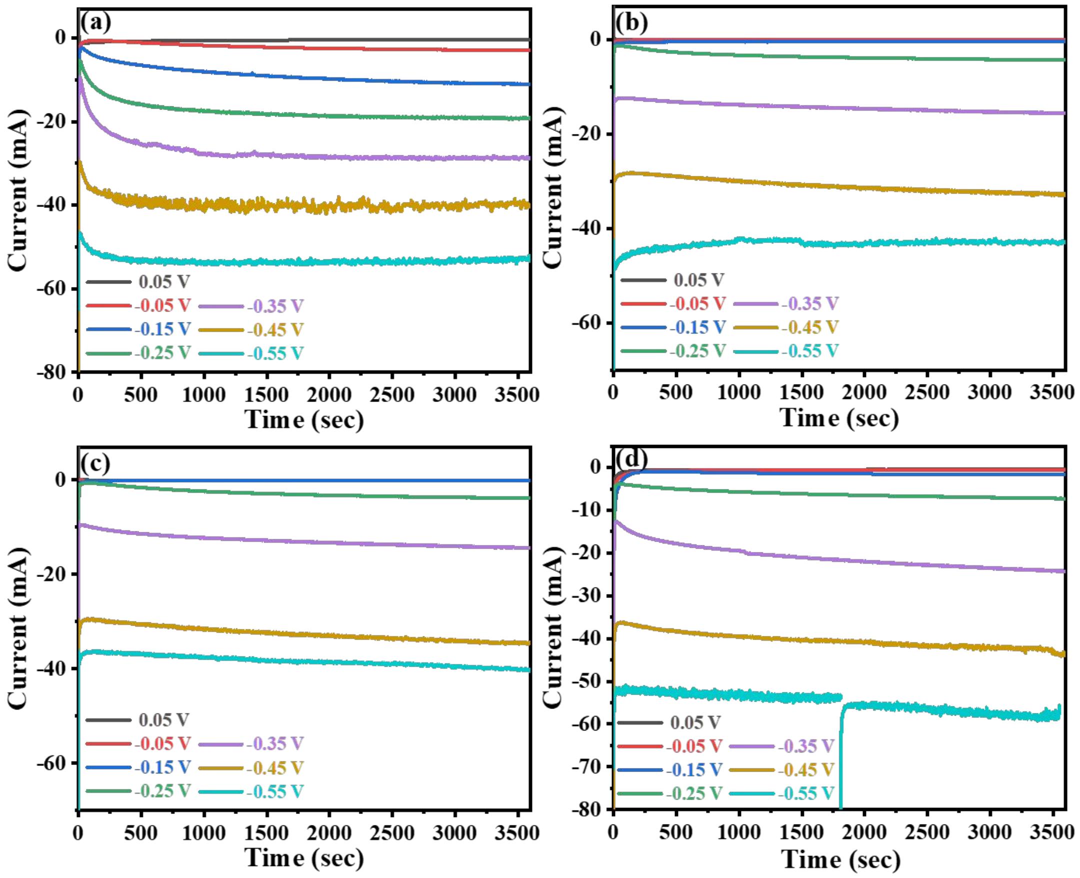

Figure S11. Chronoamperometry plots of (a) CeO_2, (b) $CeFeO_3/CeO_2$ (5 at.% Fe), (c) $CeFeO_3/CeO_2$ (25 at.% Fe), and (d) $CeFeO_3/CeO_2$ (50 at.% Fe) in 0.1 M KOH containing 0.1 M KNO_3 solution.

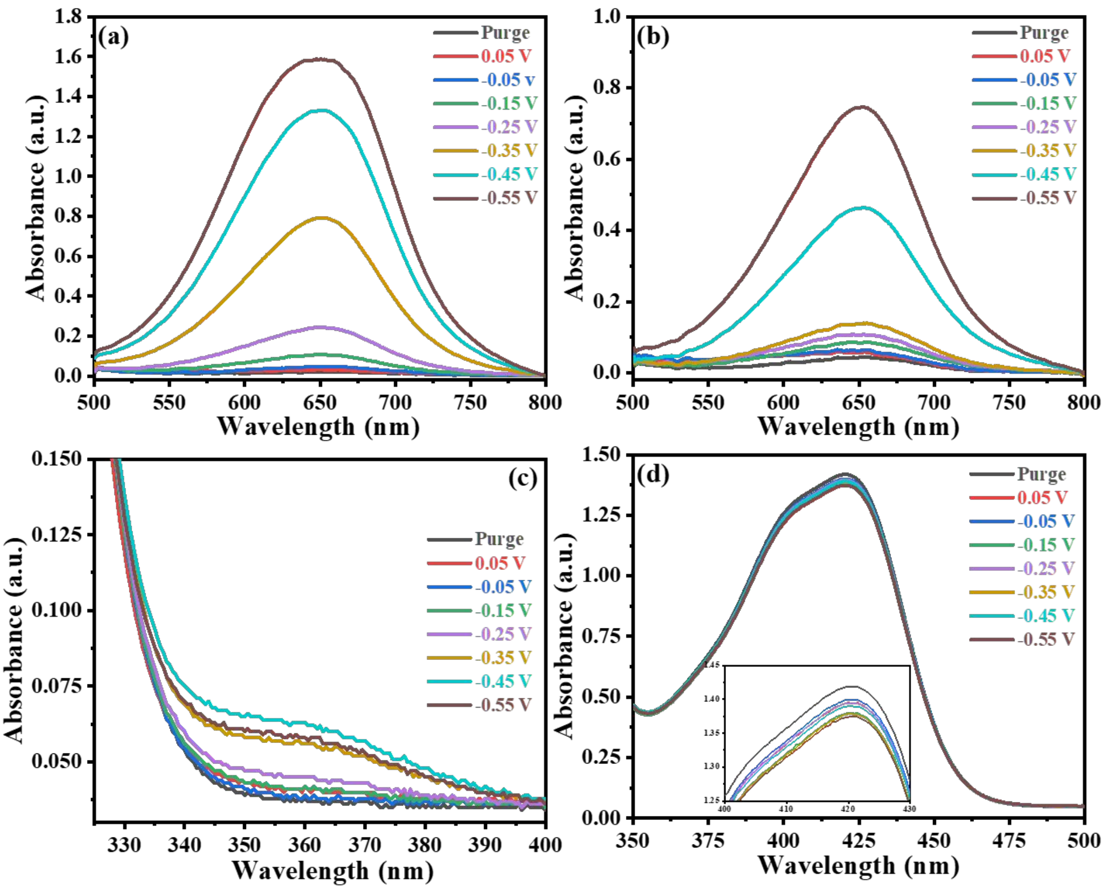

Figure S12. UV-Visible spectra of (a) ammonia in electrolyte, (b) ammonia in trap, (c) nitrite in electrolyte, and (d) hydroxylamine in electrolyte (inset shows magnified spectra) after CA on $CeFeO_3/CeO_2$ electrode in 0.1 M KOH containing 0.1 M KNO_3 solution.

Table S5. Comparison of ammonia concentration analyzed by ammonia ion selective electrode and Indophenol method.

**Potential (vs. RHE)**	**Indophenol method (µM)**		**Ion selective method (µM)**		**Deviation (%)**	
	Electrolyte	Trap	Electrolyte	Trap	Electrolyte	Trap
0.05 V	15.9	9.7	16.8	8.8	5.3	9.6
-0.05 V	35.8	12.9	33.7	12.8	5.9	0.9

-0.15 V	191.1	14.5	205.2	15.1	7.4	3.6
-0.25 V	1397.3	80.6	1485.7	87.9	6.3	8.9
-0.35 V	3960.9	396.8	3809.1	368.7	3.8	7.0
-0.45 V	5039.8	854.8	4981.2	803.2	1.2	6.0
Average deviation					5.0	6.0

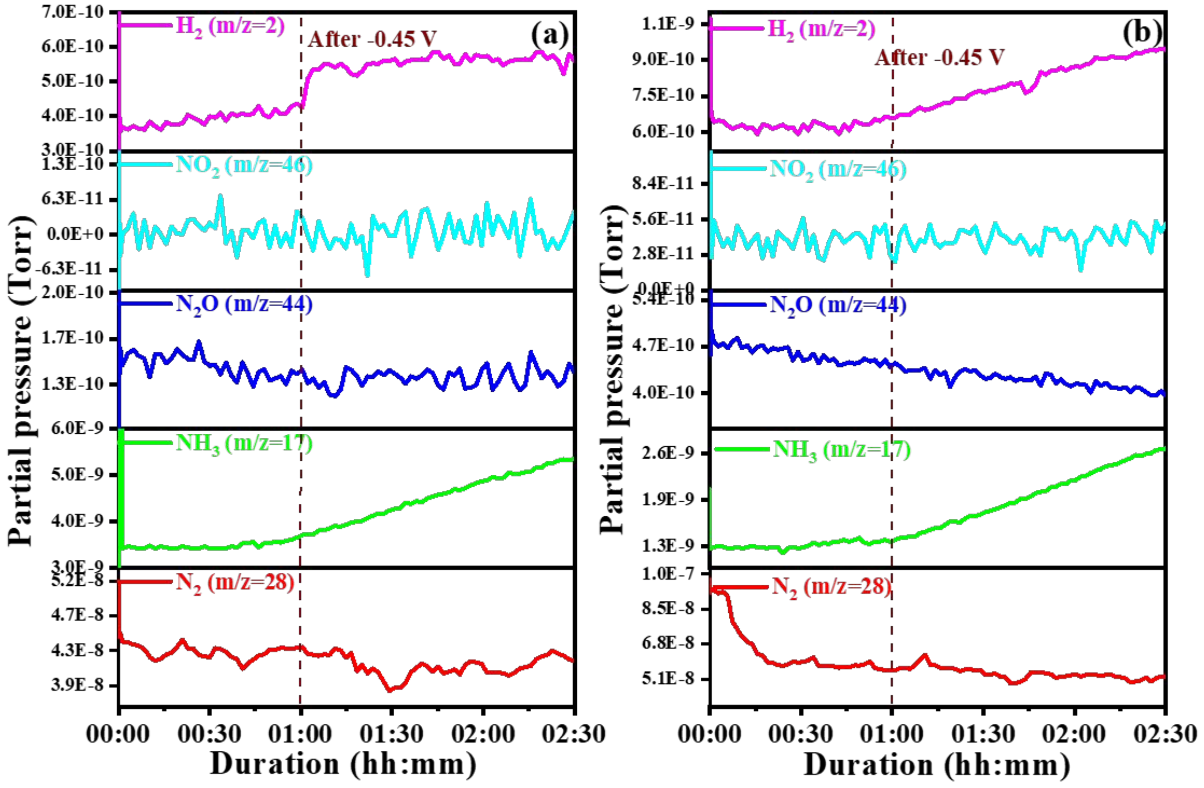

Figure S13. Online mass spectra result of (a) CeO_2, and (b) $CeFeO_3/CeO_2$ electrode in 0.1 M KOH containing 0.1 M KNO_3 solution at -0.45 V.

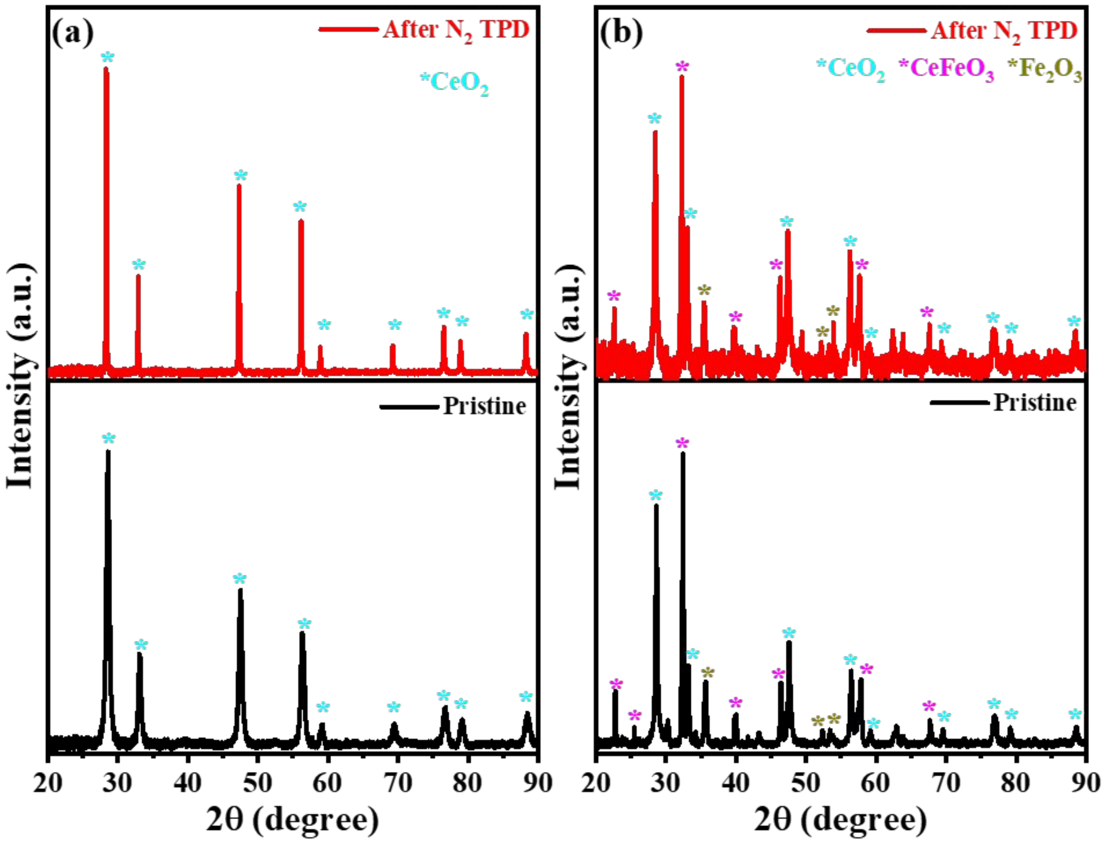

Figure S14. X-ray diffraction patterns of (a) CeO_2, and (b) $CeFeO_3/CeO_2$ after N_2 TPD analysis (25-550 ℃).

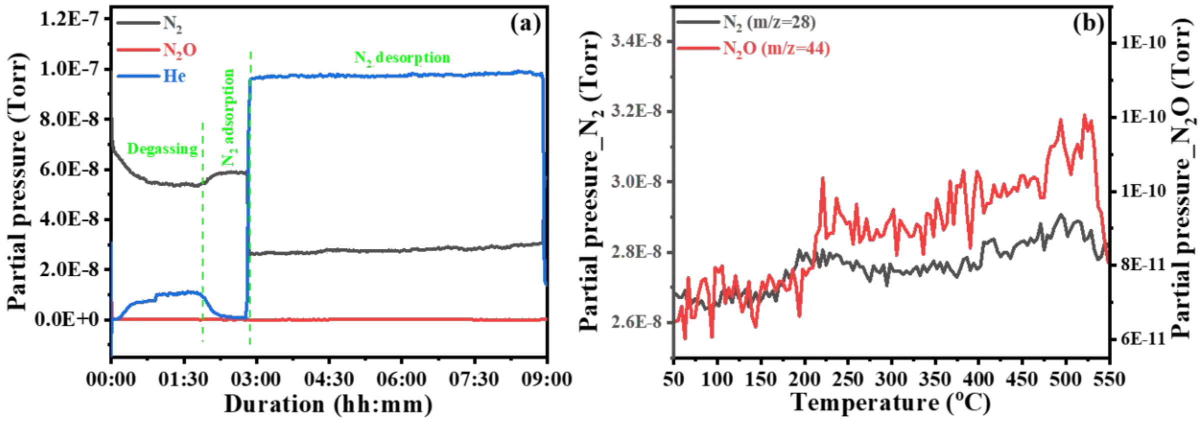

Figure S15. (a) Online mass spectra recorded during the degassing at 200 °C, nitrogen purging at 50 °C and N_2-TPD experiment of $CeFeO_3/CeO_2$ composite, and (b) magnified view of nitrogen and nitrous oxide gas in the region of N_2 desorption.

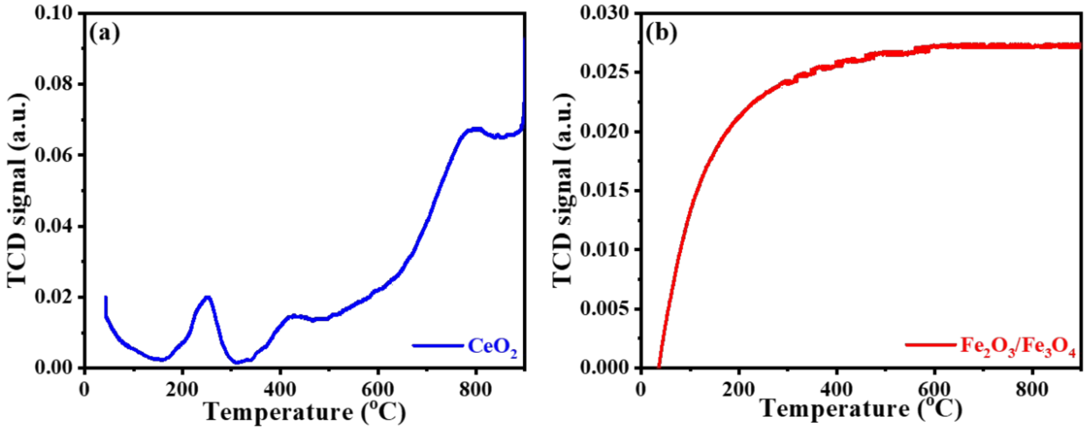

Figure S16. H_2-TPR data of (a) CeO_2, and (b) Fe_2O_3/Fe_3O_4.

Table S6. Hydrogen reactions result of $CeFeO_3/CeO_2$ catalyst from H_2-TPR analysis.

**Peak position**	**Peak area (a.u.)**	**H_2 uptake (mmol g^{-1})**
380 ℃ (CeO_2)	1.85	0.34
538 ℃ ($CeFeO_3 \rightarrow Fe_2O_3$)	8.53	1.55
598 ℃ ($Fe_2O_3 \rightarrow Fe_3O_4$)	5.44	0.99
650 ℃ ($Fe_3O_4 \rightarrow FeO$)	6.39	1.16
693 ℃ ($FeO \rightarrow Fe$)	2.98	0.54
Total H_2 uptake (mmol g^{-1})		4.57

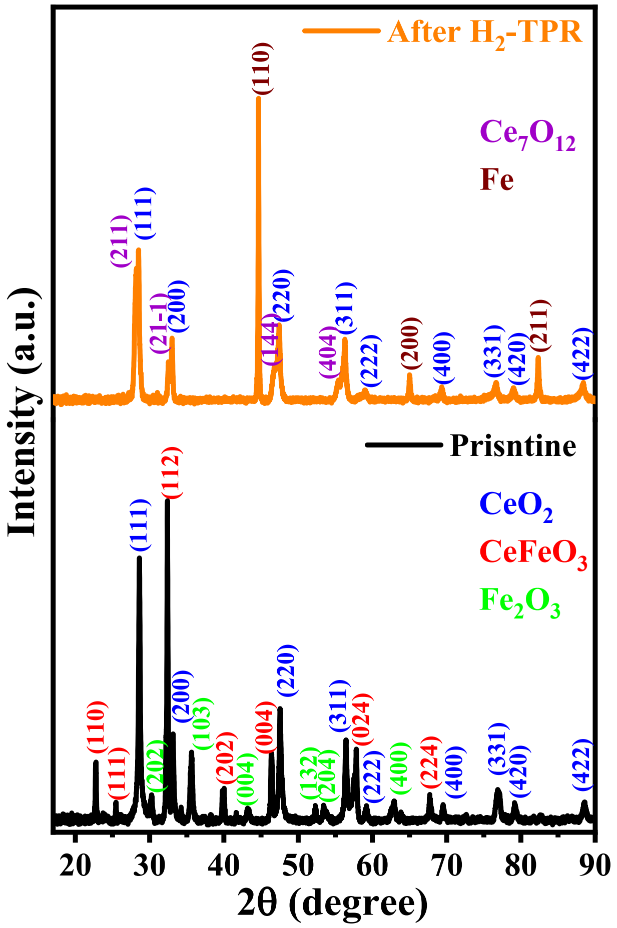

Figure S17. X-ray diffraction patterns of $CeFeO_3/CeO_2$ after H_2 TPR analysis (25-900 ℃).

Table S7. eNO_3RR performance comparison of $CeFeO_3/CeO_2$ in 0.1 M KOH with 0.1 M NO_3^- at -0.45 V vs. RHE after H_2-TPR studies.

**Experiment**	**Yield rate (μg h^{-1} cm^{-2})**			**Faradaic efficiency (%)**		
	NH_3	NO_2^-	NH_2OH	NH_3	NO_2^-	NH_2OH
**Before H_2-TPR**	3223.9	2337.5	686.8	80.1	6.7	8.2
**After H_2-TPR**	2013.8	1618.3	473.6	53.8	3.3	4.1

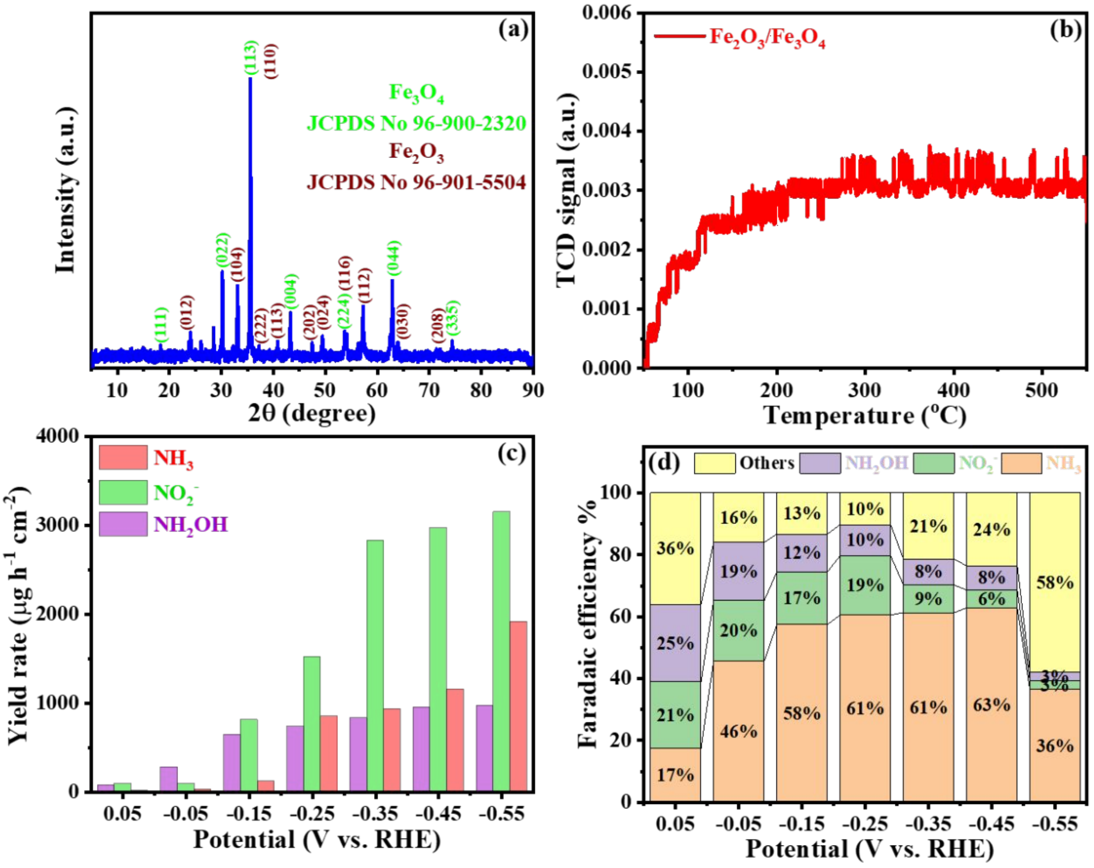

Figure S18. (a) XRD pattern, and (b) N_2-TPD profile of synthesized Fe_2O_3/Fe_3O_4; (c) Yield rate, and (d) Faradaic efficiency distribution of Fe_2O_3/Fe_3O_4 at selected applied potentials in Ar saturated 0.1 M KOH with 0.1 M NO_3^-.

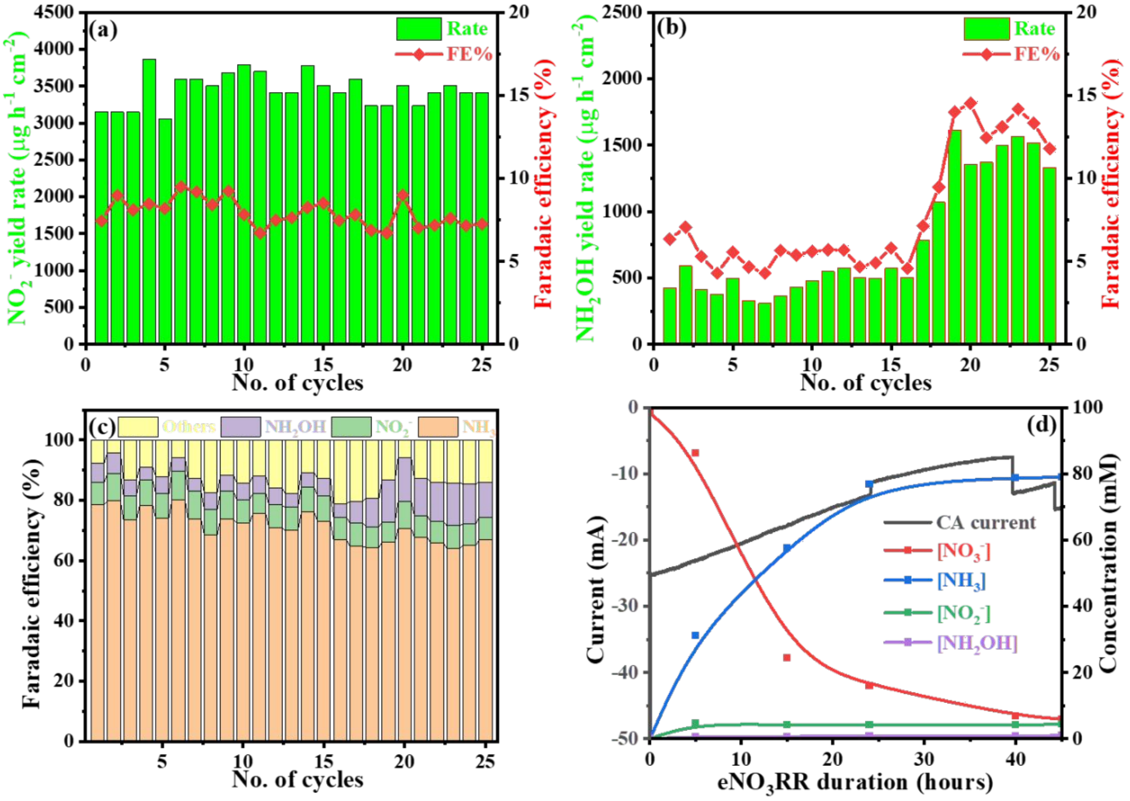

Figure S19. (a) nitrite, (b) hydroxylamine, (c) FE distribution of $CeFeO_3/CeO_2$ electrode in 25 hours of eNO_3RR cycles at -0.45 V in 0.1 M KOH with 0.1 M NO_3^-, and (d) long term eNO_3RR studies of the same.

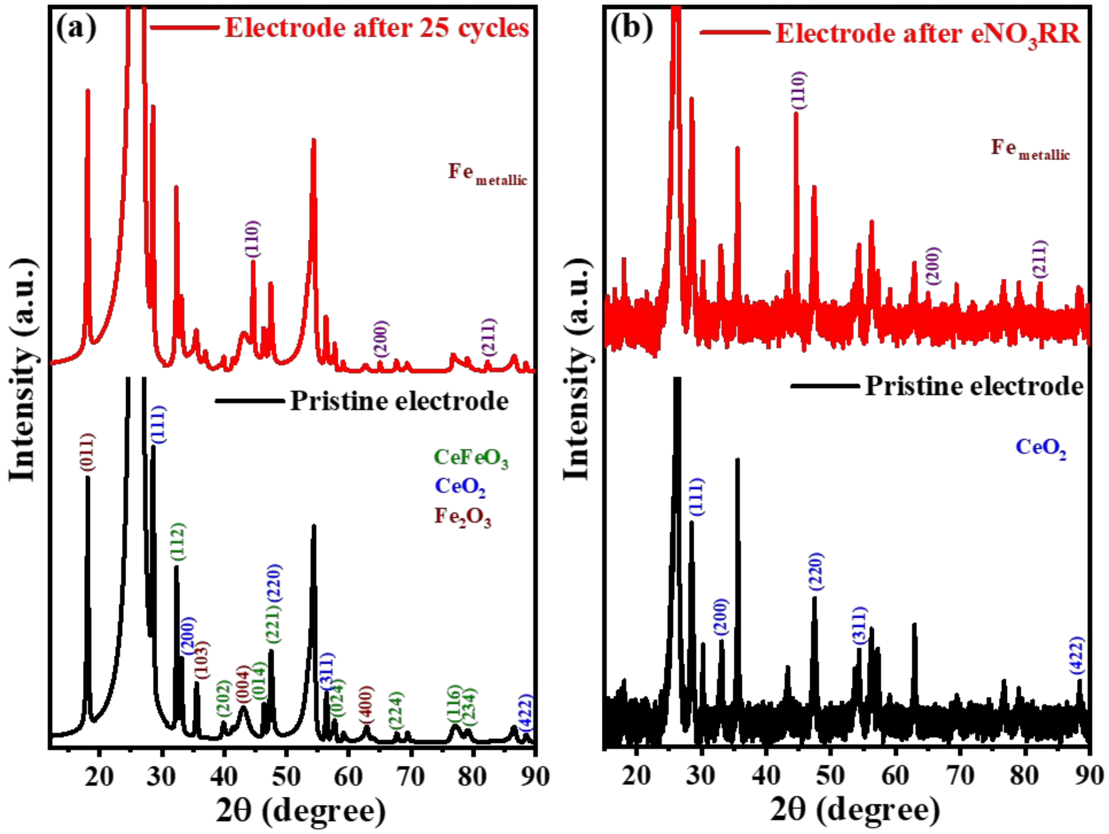

Figure S20. X-ray diffraction patterns of (a) $CeFeO_3/CeO_2$ coated electrode, and (b) Fe_2O_3/CeO_2 coated electrode after 25 hours of eNO_3RR cycles at -0.45 V in 0.1 M KOH with 0.1 M NO_3^-.

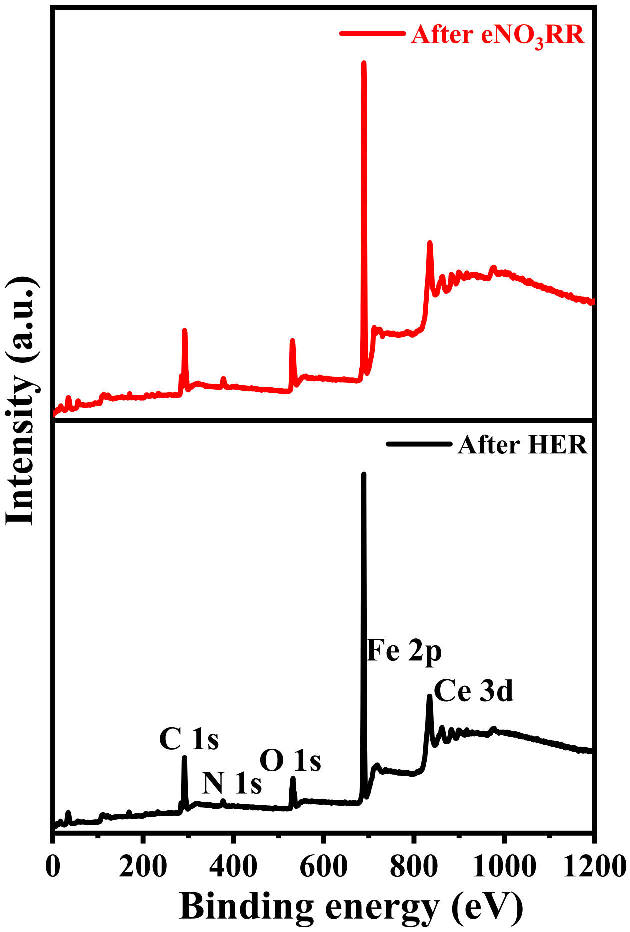

Figure S21. XPS survey spectrum of $CeFeO_3/CeO_2$ after HER and eNO_3RR at -0.45 V for 25 hours.

Table S8. Summary of XPS results of $CeFeO_3/CeO_2$ after HER and eNO_3RR at -0.45 V for 25 hours.

**Sample**	**Ce 3d (peak area %)**		**Fe 2p (peak area %)**		**O 1s (peak area %)**			
	Ce^{4+}	Ce^{2+}	Fe^{2+}	Fe^{3+}	O_L	O_V	M-O	C-O
Pristine	57.2	42.8	52.3	47.7	39.3	12.0	48.7	-
After HER	81.8	18.2	23.3	76.7	22.1	26.4	36.3	15.2

After eNO_3RR	62.8	37.2	25.7	74.3	28.9	11.9	51.5	7.7

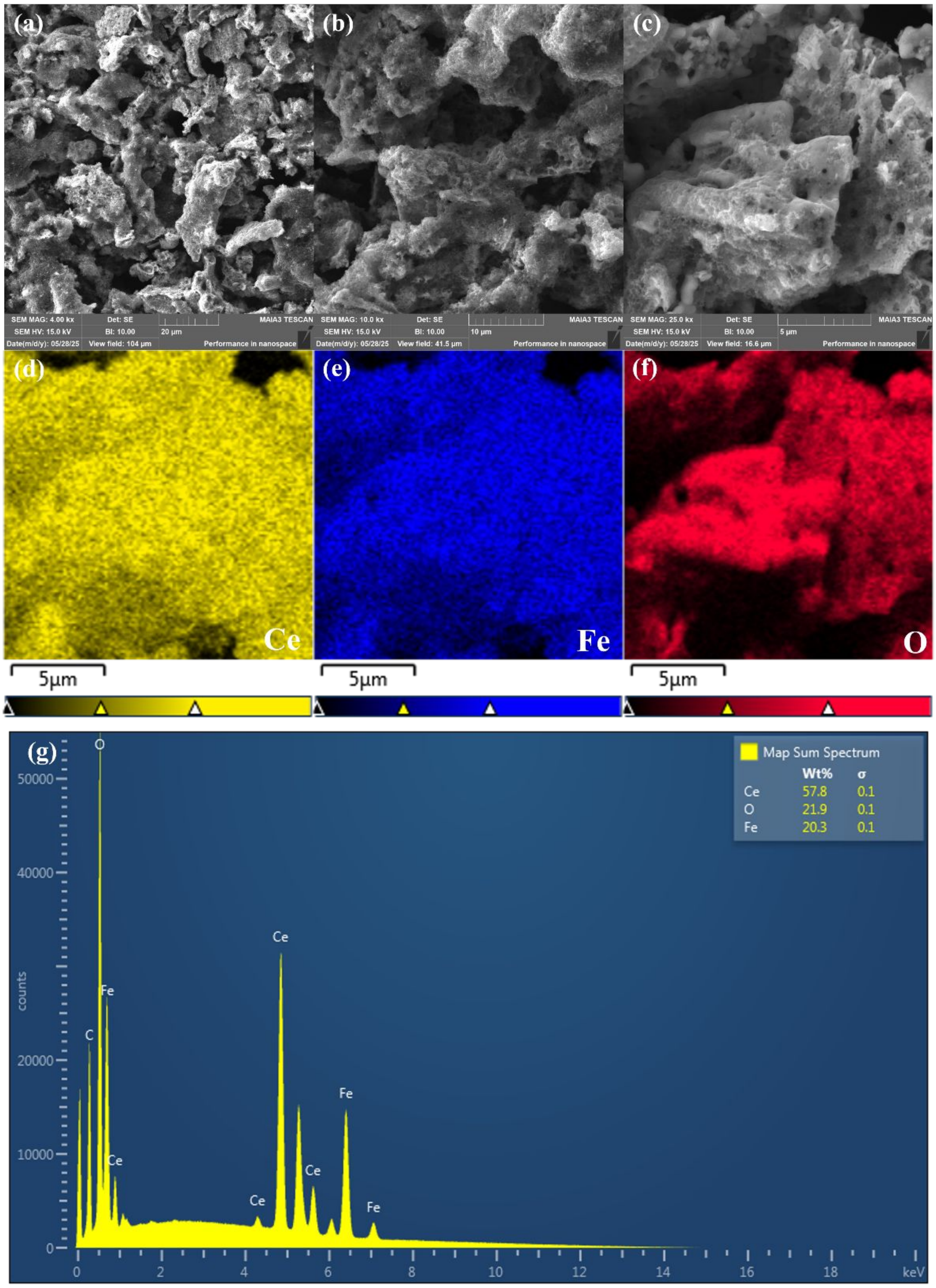

Figure S22. (a-c) Scanning electron microscopy images of $CeFeO_3/CeO_2$ coated pristine electrode at different magnifications, (d-f) EDS mapping of cerium, iron and oxygen (on Figure c), and (g) EDS spectra of the same.

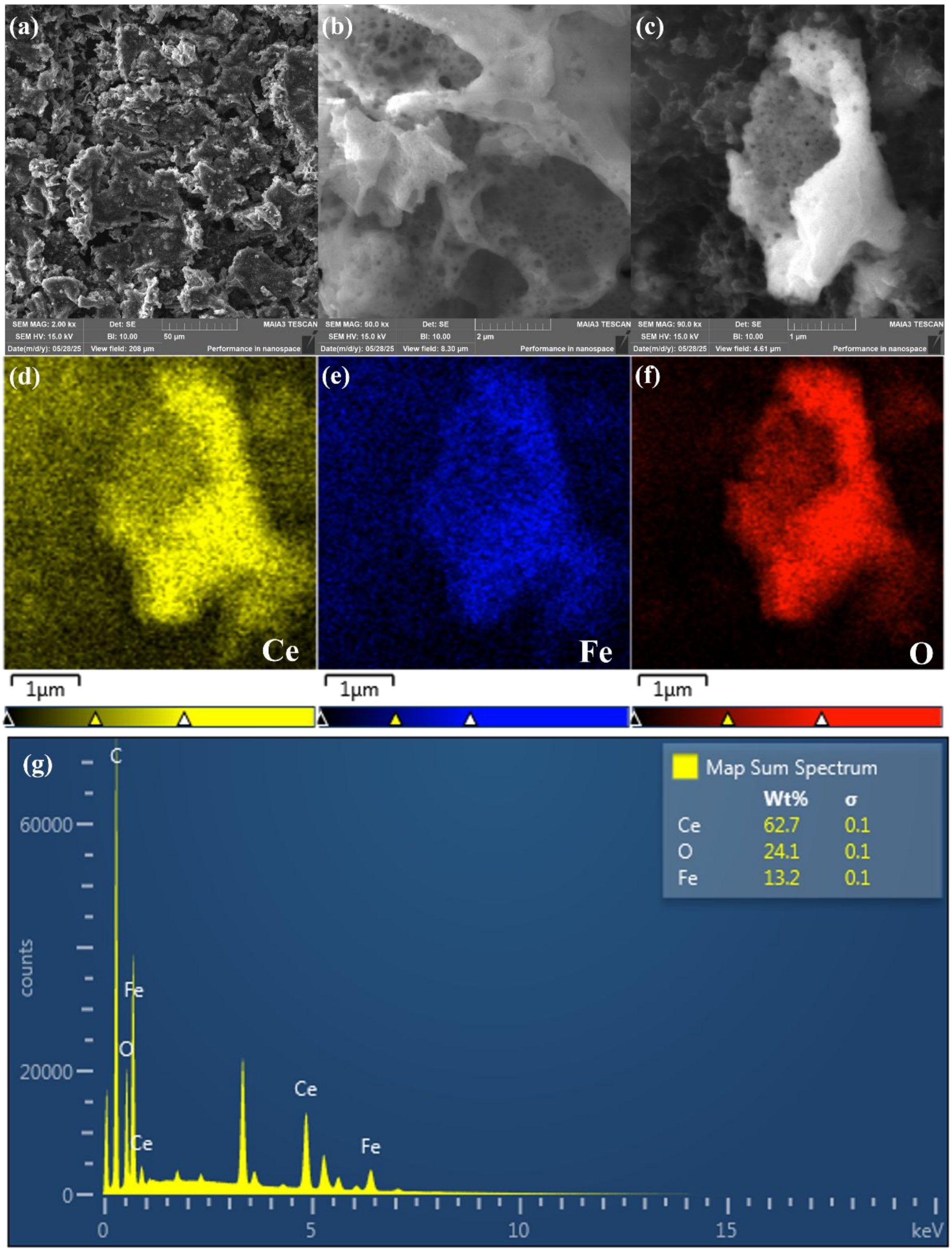

Figure S23. (a-c) Scanning electron microscopy images of $CeFeO_3/CeO_2$ coated electrode at different magnifications after stability of 25-hour eNO_3RR cycles at -0.45 V in 0.1 M KOH with 0.1 M KNO_3, (d-f) EDS mapping of cerium, iron and oxygen (on Figure c), and (g) EDS spectra of the same.

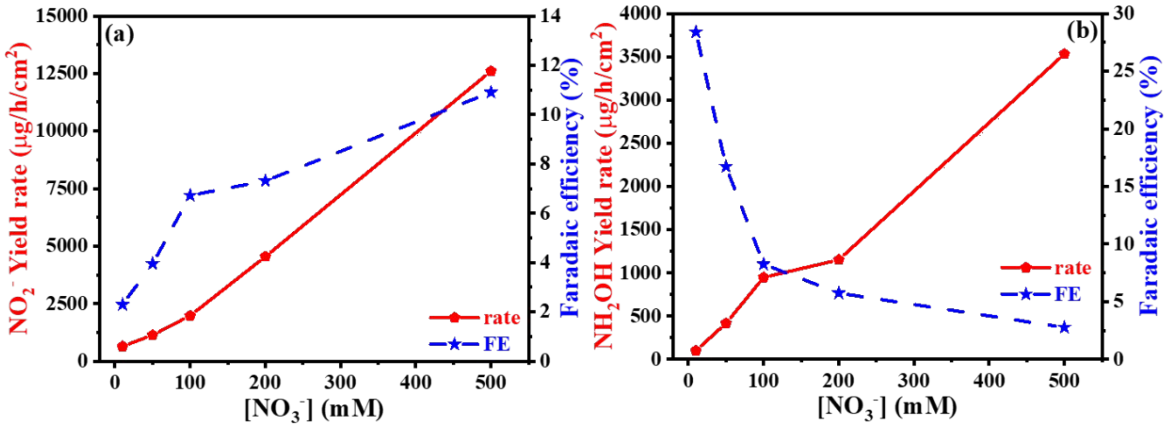

Figure S24. (a) Nitrite, and (b) hydroxylamine yield rate of $CeFeO_3/CeO_2$ in different nitrate concentrated 0.1 M KOH electrolyte at -0.45 V.

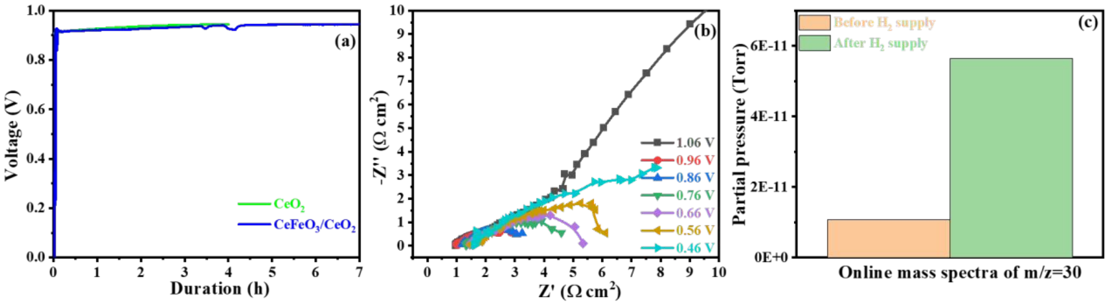

Figure S25. (a) OCV, (b) PEIS recorded at different potentials, and (c) Online mass spectra measurements (at OCV of 1.06) of H_2-NO_3^- fuel cell using $CeFeO_3/CeO_2$ as cathode and Pt/C anode.

Table S9. Fuel cell performance comparison of $CeFeO_3/CeO_2$ with recently reported catalysts.

S. No	Fuel cell Type	Catalyst	OCV (V)	Power density (mW cm^{-2})	Current density (mA cm^{-2})	Reference
1	Zn-NO_3^-	Fe_2TiO_5 nanofibers	1.50	5.6	10.5	[1]
2	Zn-NO_2^-	C/Co_3O_4	1.45	6.0	15.3	[2]

3	Zn-NO_3^-	Fe/Ni_2P	1.22	3.2	3.1	[3]
4	Zn-NO_3^-	Ni1Cu-SAA	1.51	12.7	30.4	[4]
5	Zn-NO_3^-	Metastable phase-Cu	1.27	7.6	15.2	[5]
6	Zn-NO_3^-	Cu nanowires	0.93	14.1	40.2	[6]
7	Zn-NO_3^-	Ru/β-$Co(OH)_2$	1.48	29.9	50.5	[7]
8	Zn-NO_3^-	Ru/Ni hydroxide	1.20	23.3	100.6	[8]
9	Zn-NO_3^-	Cu @ Cu foam	1.40	3.9	8.2	[9]
10	Zn-NO_3^-	CuPd/CuO @ NF	1.04	53.7	170.5	[10]
11	Zn-NO_3^-	Ni_1Co_2/CC	1.02	5.1	20.5	[11]
10	Alkaline-acid hybrid Zn-NO_3^-	TiO_2/FePc	1.99	91.4	160.5	[12]
11	Urea – NO_3^-	Ni@NiO-Cu@CuO/NCS	0.72	22.5	141.0	[13]
**12**	**H_2-NO_3^-**	**$CeFeO_3/CeO_2$**	**0.91**	**19.2**	**29.7**	**This work**

References

(1) Du, H.; Guo, H.; Wang, K.; Du, X.; Beshiwork, B. A.; Sun, S.; Luo, Y.; Liu, Q.; Li, T.; Sun, X. Durable Electrocatalytic Reduction of Nitrate to Ammonia over Defective Pseudobrookite Fe2TiO5 Nanofibers with Abundant Oxygen Vacancies. *Angewandte Chemie - International Edition* **2023**, *62* (5). https://doi.org/10.1002/anie.202215782.

(2) Zhang, R.; Zhang, S.; Guo, Y.; Li, C.; Liu, J.; Huang, Z.; Zhao, Y.; Li, Y.; Zhi, C. A Zn-Nitrite Battery as an Energy-Output Electrocatalytic System for High-Efficiency Ammonia Synthesis Using Carbon-Doped Cobalt Oxide Nanotubes. *Energy Environ Sci* **2022**, *15* (7), 3024–3032. https://doi.org/10.1039/d2ee00686c.

(3) Zhang, R.; Guo, Y.; Zhang, S.; Chen, D.; Zhao, Y.; Huang, Z.; Ma, L.; Li, P.; Yang, Q.; Liang, G.; Zhi, C. Efficient Ammonia Electrosynthesis and Energy Conversion through a Zn-Nitrate Battery by Iron Doping Engineered Nickel Phosphide Catalyst. *Adv Energy Mater* **2022**, *12* (13). https://doi.org/10.1002/aenm.202103872.

(4) Cai, J.; Wei, Y.; Cao, A.; Huang, J.; Jiang, Z.; Lu, S.; Zang, S. Q. Electrocatalytic Nitrate-to-Ammonia Conversion with ~100% Faradaic Efficiency via Single-Atom Alloying. *Appl Catal B* **2022**, *316*. https://doi.org/10.1016/j.apcatb.2022.121683.

(5) Wen, W.; Yan, P.; Sun, W.; Zhou, Y.; Yu, X. Y. Metastable Phase Cu with Optimized Local Electronic State for Efficient Electrocatalytic Production of Ammonia from Nitrate. *Adv Funct Mater* **2023**, *33* (6). https://doi.org/10.1002/adfm.202212236.

(6) Jiang, H.; Chen, G. F.; Savateev, O.; Xue, J.; Ding, L. X.; Liang, Z.; Antonietti, M.; Wang, H. Enabled Efficient Ammonia Synthesis and Energy Supply in a Zinc–Nitrate Battery System by Separating Nitrate Reduction Process into Two Stages. *Angewandte Chemie - International Edition* **2023**, *62* (13). https://doi.org/10.1002/anie.202218717.

(7) Zhu, W.; Yao, F.; Wu, Q.; Jiang, Q.; Wang, J.; Wang, Z.; Liang, H. Weakened D-p Orbital Hybridization in in Situ Reconstructed Ru/β-Co(OH)2 Heterointerfaces for Accelerated Ammonia Electrosynthesis from Nitrates. *Energy Environ Sci* **2023**, *16* (6), 2483–2493. https://doi.org/10.1039/d3ee00371j.

(8) Zhou, F.; Sun, C. Nitrate-to-Ammonia Conversion on Ru/Ni Hydroxide Hybrid through Zinc-Nitrate Fuel Cell. *Small* **2022**, *18* (21). https://doi.org/10.1002/smll.202200436.

(9) Ma, P.; Lu, L.; Wang, Q.; Bi, R.; Chen, F.; Tang, Q.; Ma, X. Surface-Modified Copper Foam for Nitrate-to-Ammonia and Zinc-Nitrate Fuel Cell Catalysis. *Mater Res Bull* **2025**, *181*. https://doi.org/10.1016/j.materresbull.2024.113079.

(10) Li, J.; Liu, L.; Huang, S.; Wang, H.; Tang, Y.; Zhang, C.; Du, F.; Ma, R.; Li, C.; Guo, C. Nanoflower-Like CuPd/CuO Heterostructure for an Energy-Output Electrocatalytic System Coupling Ammonia Electrosynthesis and Zinc-Nitrate Battery. *Adv Funct Mater* **2025**. https://doi.org/10.1002/adfm.202501527.

(11) Shen, Y.; Dou, Y.; Sun, L.; Huo, L.; Zhao, H. Work Function-Induced Electronic Modulation in NiCo Alloy for Electrochemical Nitrate Reduction. *Inorg Chem* **2025**. https://doi.org/10.1021/acs.inorgchem.5c00362.

(12) Zhang, R.; Li, C.; Cui, H.; Wang, Y.; Zhang, S.; Li, P.; Hou, Y.; Guo, Y.; Liang, G.; Huang, Z.; Peng, C.; Zhi, C. Electrochemical Nitrate Reduction in Acid Enables High-Efficiency Ammonia Synthesis and High-Voltage Pollutes-Based Fuel Cells. *Nat Commun* **2023**, *14* (1). https://doi.org/10.1038/s41467-023-43897-6.

(13) Nangan, S.; Ding, Y.; Alhakemy, A. Z.; Liu, Y.; Wen, Z. Hybrid Alkali-Acid Urea-Nitrate Fuel Cell for Degrading Nitrogen-Rich Wastewater. *Appl Catal B* **2021**, *286*. https://doi.org/10.1016/j.apcatb.2021.119892.
